# Mesenchymal stem cells attenuate liver fibrosis by targeting Ly6C^hi/lo^ macrophages through activating the cytokine-paracrine and apoptotic pathways

**DOI:** 10.1038/s41420-021-00584-z

**Published:** 2021-09-13

**Authors:** Yuan-hui Li, Shuang Shen, Tong Shao, Meng-ting Jin, Dong-dong Fan, Ai-fu Lin, Li-xin Xiang, Jian-zhong Shao

**Affiliations:** 1 College of Life Sciences, Key Laboratory for Cell and Gene Engineering of Zhejiang Province, Zhejiang University, Hangzhou, People’s Republic of China; 2 Laboratory for Marine Biology and Biotechnology, Qingdao National Laboratory for Marine Science and Technology, Qingdao, People’s Republic of China

**Keywords:** Monocytes and macrophages, Mesenchymal stem cells

## Abstract

Mesenchymal stem cell (MSC) therapy has become a promising treatment for liver fibrosis due to its predominant immunomodulatory performance in hepatic stellate cell inhibition and fibrosis resolution. However, the cellular and molecular mechanisms underlying these processes remain limited. In the present study, we provide insights into the functional role of bone marrow-derived MSCs (BM-MSCs) in alleviating liver fibrosis by targeting intrahepatic Ly6C^hi^ and Ly6C^lo^ macrophage subsets in a mouse model. Upon chronic injury, the Ly6C^hi^ subset was significantly increased in the inflamed liver. Transplantation of BM-MSCs markedly promoted a phenotypic switch from pro-fibrotic Ly6C^hi^ subset to restorative Ly6C^lo^ subpopulation by secreting paracrine cytokines IL-4 and IL-10 from the BM-MSCs. The Ly6C^hi^/Ly6C^lo^ subset switch significantly blocked the source of fibrogenic TGF-β, PDGF, TNF-α, and IL-1β cytokines from Ly6C^hi^ macrophages. Unexpectedly, BM-MSCs experienced severe apoptosis and produced substantial apoptotic bodies in the fibrotic liver during the 72 h period of transplantation. Most apoptotic bodies were engulfed by Ly6C^lo^ macrophages, and this engulfment robustly triggered MMP12 expression for fibrosis resolution through the PtdSer-MerTK-ERK signaling pathway. This paper is the first to show previously unrecognized dual regulatory functions of BM-MSCs in attenuating hepatic fibrosis by promoting Ly6C^hi^/Ly6C^lo^ subset conversion and Ly6C^lo^ macrophage restoration through secreting antifibrogenic-cytokines and activating the apoptotic pathway.

## Introduction

Hepatic fibrosis is a dynamic process characterized by excessive accumulation of extracellular matrix (ECM), mainly fibrillar collagens resulting from ongoing chronic liver injury and inflammation of many etiologies [1]. Activation of hepatic stellate cells (HSCs) into proliferative and fibrogenic myofibroblasts is well established as a central driver of liver fibrogenesis in experimental and human hepatic injury [2, 3]. This process is regulated by numerous extracellular signals from various resident and inflammatory cells, among which bone marrow monocyte-derived macrophages were the major modulators [4]. In mice, the circulating monocytes derived from bone marrow are composed of at least Ly6C^hi^ and Ly6C^lo^ subsets. These two subsets are characterized by distinct CX3CR1^mid^CCR2^+^Ly6C^hi^ and CX3CR1^hi^CCR2^-^Ly6C^lo^ phenotypes [5–7]. Two functional equivalents with CD14^hi^CD16^lo^ and CD14^lo^CD16^hi^ phenotypes were preliminarily identified in humans [8–10]. In response to injury, the Ly6C^hi^ monocytes quickly respond to inflammatory signals and migrate to the inflamed liver, wherein they differentiate into macrophages. The intrahepatic Ly6C^hi^ macrophages are highly inflammatory and fibrogenic, whereas the Ly6C^lo^ cells are considered alternative macrophages that can dampen inflammation and diminish liver fibrosis [11]. Ly6C^hi^ macrophages promote HSC activation by producing a variety of cytokines and chemokines, including TGF-β, PDGF, TNF-α, IL-1β, MCP1, CCL3, and CCL5 [4]. Ly6C^lo^ macrophages promote fibrosis resolution by secreting matrix metalloproteinases (MMPs) such as MMP12 and MMP13 and upregulating TNF-related apoptosis-inducing ligand (TRAIL) that induces HSC apoptosis [12]. The Ly6C^hi^ monocytes/macrophages express high levels of CCR1 and CCR2, which promote the infiltration and accumulation of Ly6C^hi^ cells in the inflamed liver [13]. Instead, Ly6C^lo^ monocytes/macrophages display enhanced expression of CX3CR1, which inhibits inflammatory properties in macrophages by binding its ligand CX3CL1 [14].

Although mechanisms underlying hepatic fibrogenesis have been increasingly clarified, the development of effective therapies for the regression of fibrosis remains a challenge. In the past decade, transplantation of exogenous bone marrow-derived mesenchymal stem cells (BM-MSCs) has become a promising cytotherapy for treating liver fibrosis due to the predominant role of BM-MSCs in HSC inhibition and fibrosis resolution [15, 16]. Multiple immunomodulatory factors secreted from BM-MSCs were found to inhibit proliferation or induce apoptosis of HSCs, including IL-10, HGF, TGF-β, TNF-α, PGE-2, IDO, and NO [17–19]. Additionally, our previous studies found that BM-MSCs attenuate HSC activation and liver fibrosis by inhibiting Delta-like1 expression and paracrine from hepatocytes [20]. Here, we demonstrated that BM-MSCs attenuate HSC activation and hepatic fibrosis by targeting Ly6C^hi^ and Ly6C^lo^ macrophages through activating the antifibrogenic-cytokine paracrine and apoptotic pathways, thereby uncovering a previously unrecognized mechanism underlying MSC-mediated therapeutic options in liver repair.

## Results

### BM-MSCs attenuate liver fibrosis by promoting Ly6C^hi^/Ly6C^lo^ macrophage conversion

**Fig. 1:**
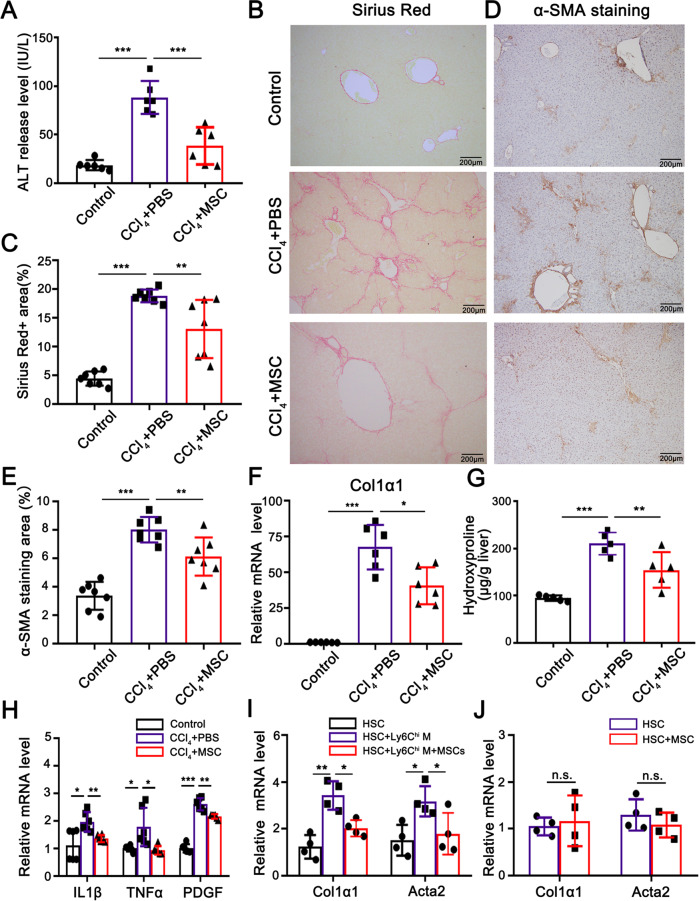
BM-MSC transplantation attenuated CCl_4_-induced inflammatory levels and liver fibrosis. **A** ALT release level in the serum of normal control mice and fibrotic mice transplanted with BM-MSCs or PBS (control). Serum samples were collected at 2 weeks post-transplantation (*n* = 6). **B**, **C** Liver tissue sections were stained with Sirius Red (**B**), and the degree of liver fibrosis was semi-quantified by Image software (**C**) (*n* = 7). Scale bars, 200 μm. **D**, **E** Immunohistochemistry staining of α-SMA in each group (**D**) and α-SMA^+^ areas were semi-quantified by Image software (**E**) (*n* = 7). Scale bars, 200 μm. **F** Quantification of *col1α1* mRNA expression levels related to GAPDH by quantitative real-time PCR (*n* = 6). **G** Examination of hydroxyproline contents in livers at 2 weeks post-transplantation of BM-MSCs (*n* = 5). **H** Relative expression levels of *il-1β*, *tnf-α*, and *pdgf* mRNAs in livers of normal control and fibrotic mice transplanted with BM-MSCs or PBS examined by quantitative real-time PCR (*n* = 6). **I** Relative expression levels of *col1α1* and *acta2* mRNAs in HSCs, HSCs cocultured with Ly6C^hi^ macrophages, and HSCs cocultured with Ly6C^hi^ macrophages plus BM-MSCs examined by quantitative real-time PCR (*n* = 4). Ly6C^hi^ M is the abbreviation of Ly6C^hi^ macrophages. **J** Relative expression levels of *col1α1* and *acta2* mRNAs in HSCs cocultured with BM-MSCs or without BM-MSCs (control) were examined by quantitative real-time PCR (*n* = 4). Bars = means ± SD. Statistical evaluation of two groups was performed using an independent Student *t*-test. Statistical evaluation of multiple groups was performed using one-way ANOVA with posthoc LSD test; n.s. *p* > 0.05, *p < 0.05, **p < 0.01, ***p < 0.001.

**Fig. 2:**
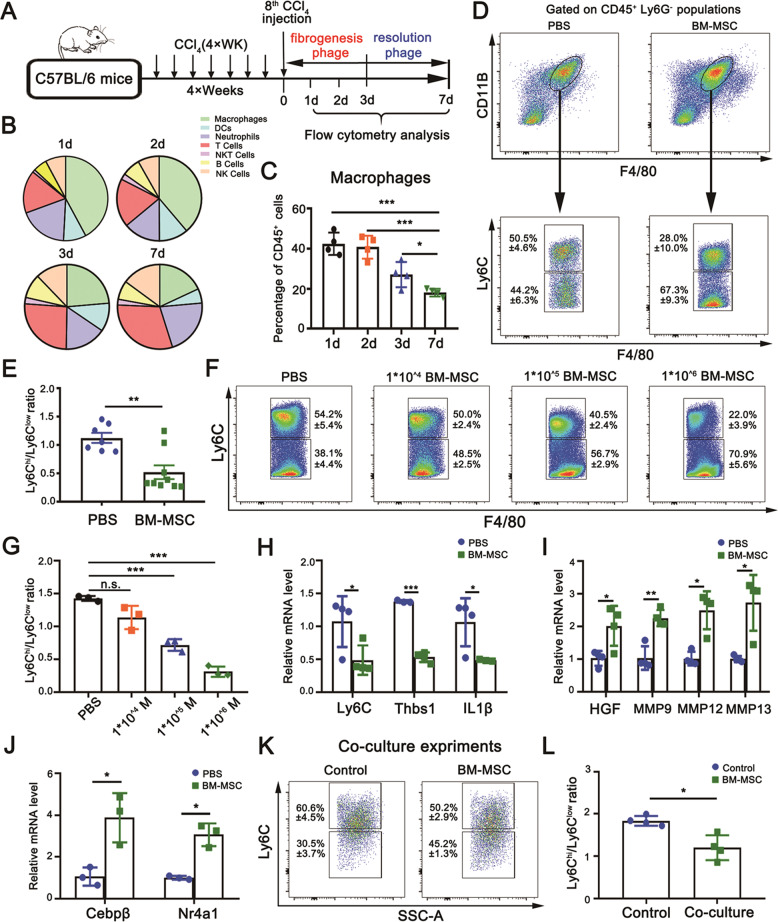
Evaluation of the contribution of BM-MSCs to the conversion of Ly6C^hi^ to Ly6C^lo^ macrophages. **A** Schematic of the work model. C57BL/6 J mice were i.v. injected with CCl_4_ (1 mg/kg) twice a week for 4 weeks, followed by FCM analysis to detect different immunocytes at 1, 2, 3 (fibrogenesis stage), and 7 days (resolution stage) after the final CCl_4_ injection. **B** The proportion of various immunocytes in fibrotic livers at fibrogenesis and resolution after the final CCl_4_ injection analyzed by FCM. **C** FCM analysis showing a strong response of the intrahepatic macrophage population to CCl_4_ injury at the early fibrogenesis stage (*n* = 4). **D**, **E** Alteration in the ratio of Ly6C^hi^/Ly6C^lo^ macrophages in fibrotic livers 7 days post-BM-MSC transplantation by FCM analysis (*n* = 7 and *n* = 9). **F**, **G** FCM analysis showed that the conversion of Ly6C^hi^ to Ly6C^lo^ macrophages in fibrotic livers was conducted in a dose-dependent manner in response to BM-MSC transplantation with different concentrations (0, 1 × 10^4^, 1 × 10^5^, and 1 × 10^6^; *n* = 3). In figure (**G**), M is an abbreviation of experimental groups with BM-MSC transplantation. **H**–**J** Quantitative real-time PCR analysis for the transcriptional expression levels of pro-inflammation markers (**H**), pro-resolution markers (**I**), and transcription factors for Ly6C^lo^ monocyte differentiation (**J**) in CD11B^hi^F4/80^int^ macrophages sorted from fibrotic livers at 24 h post-final CCl_4_ injection (*n* = 4 and *n* = 3). **K**, **L** FCM analysis for the phenotypic switch from Ly6C^hi^ to Ly6C^lo^ macrophages by coculturing BM-MSCs with equivalent CD11B^hi^F4/80^int^ macrophages isolated from the fibrotic liver for 36 h (n = 4). Bars = means ± SD. Statistical evaluation of two groups was performed using an independent Student *t*-test. Statistical evaluation of multiple groups was performed using one-way ANOVA with posthoc LSD test; n.s. *p* > 0.05, *p < 0.05, **p < 0.01, ***p < 0.001.

A primary bone marrow-derived mesenchymal stem cell (BM-MSC) line with typical mesenchymal lineage markers and differentiation potency was transplanted in a CCl_4_-induced fibrotic C57BL/6 J mouse model (Fig. S1); and the transplantation remarkably attenuated liver fibrosis by decreasing serum alanine aminotransferase (ALT) level, intrahepatic collagen deposition and HSC activation (Fig. 1A–G). This outcome was accompanied by a robust reduction in profibrogenic cytokines including IL-1β, TNF-α, and PDGF derived from pro-fibrotic cells such as Ly6C^hi^ macrophages (Fig. 1H). Quiescent and activated HSCs isolated from mouse livers were used for in vitro coculture experiments (Fig. S2). Coculture of quiescent HSCs with Ly6C^hi^ macrophages promoted HSC activation, as examined by the upregulation of *α-SMA* and *col1α1* mRNAs; whereas coculture of quiescent HSCs with Ly6C^hi^ macrophages plus BM-MSCs impaired such an activation. Additionally, coculture of activated HSCs isolated from the fibrotic liver with BM-MSCs alone did not affect the expression of *α-SMA* and *col1α1* mRNAs (Fig. 1I, J). These results suggest that BM-MSCs indirectly suppressed HSC activation through inhibiting Ly6C^hi^ macrophages. Actually, Ly6C^hi^ macrophages were the most prevalent and significantly increased population in the fibrotic liver (Figs. 2B, C, S3, S4). The Ly6C^hi^ macrophages exhibited a CD45^+^Ly6G^−^CD11B^hi^F4/80^int^CX3CR1^mid^CCR2^+^Ly6C^hi^ phenotype, suggesting the infiltration of circulating Ly6C^hi^ monocytes into the inflamed liver as previously believed. After BM-MSC transplantation, the ratio of Ly6C^hi^/Ly6C^lo^ macrophages decreased by 51% compared with that of the mock PBS-administered control group (Figs. 2D, E, S6). This promotion was enhanced with the increase in the cells administered (Fig. 2F, G). Other immunocytes had no evident changes after BM-MSC infusion (Fig. S5). The Ly6C^hi^/Ly6C^lo^ macrophage conversion was also evaluated by the upregulation of two regulatory genes (*nr4a1* and *cebpβ*) and disparate transcriptional alteration of Ly6C^hi^ (*ly6c*, *thbs1,* and *il1β*) and Ly6C^lo^ (*hgf*, *mmp9*, *mmp12,* and *mmp13*) marker genes in CD45^+^Ly6G^−^CD11B^hi^F4/80^int^ inflammatory macrophages (named CD11B^hi^F4/80^int^) sorted from fibrotic liver transplanted with BM-MSCs by FACS (Fig. 2H, I, J). Additionally, the conversion was further detected by incubating CD11B^hi^F4/80^int^ macrophages with BM-MSCs in vitro (Fig. 2K, L). These data indicated that BM-MSCs could directly promote the phenotypic switch from Ly6C^hi^ to Ly6C^lo^ macrophages in the fibrotic liver, which led to the conversion of macrophages from a pro-inflammatory state to pro-resolving status.

### BM-MSCs hardly prevent the recruitment of Ly6C^hi^ monocytes to injured liver

**Fig. 3:**
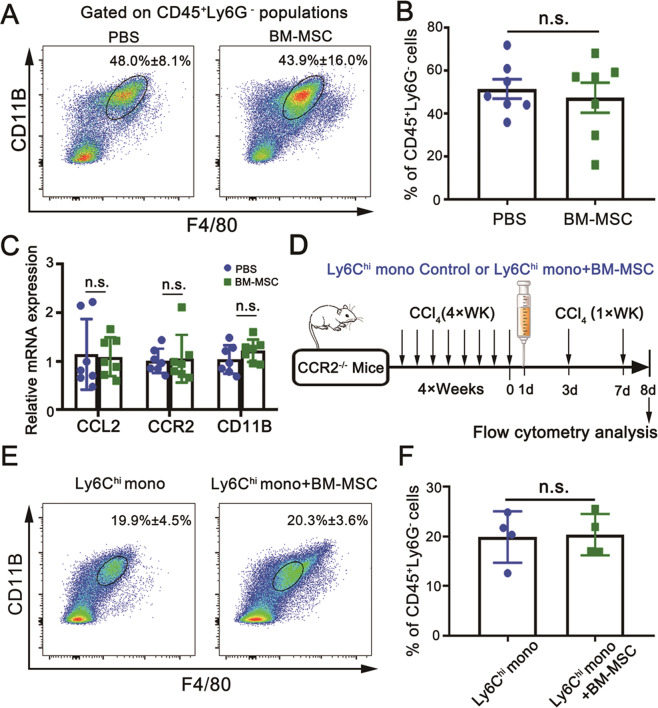
Evaluation of the effect of BM-MSCs on Ly6C^hi^ monocyte recruitment. **A**, **B** Examination of the proportion of CD11B^hi^F4/80^int^ macrophages in livers at 24 h post-final CCl_4_ injection by FCM analysis (*n* = 7). CCl_4_-induced fibrotic mice were infused with 1 × 10^6^ BM-MSCs or PBS (control) at 24 h post-8^th^ CCl_4_ injection, and mice were continually injected with CCl_4_ for another week. **C** Quantitative real-time PCR analysis for the transcriptional expression levels of genes related to chemotaxis (*n* = 7). **D** Schematic of the working model for in vivo adoptive transfer assay by using a CCR2^−/−^ mouse. The CCR2^−/−^ mice were i.v. injected with CCl_4_ (1 mg/kg) twice a week for 4 weeks. Thereafter, 5 × 10^5^ Ly6C^hi^ monocytes were transferred into CCR2^−/−^ fibrotic mice with or without equivalent BM-MSCs at 24 h post-final CCl_4_ injection. FCM analysis was performed at 7 days after adoptive transfer. Ly6C^hi^ mono is the abbreviation of Ly6C^hi^ monocytes. **E**, **F** Examination of the proportion of intrahepatic CD11B^hi^F4/80^int^ macrophages from different transfer groups by FCM analysis (*n* = 4). Bars = means ± SD. Statistical evaluation of two groups was performed using independent Student *t*-test; n.s. *p* > 0.05.

Given that bone marrow-derived Ly6C^hi^ monocytes that highly express CCR2 can be recruited from peripheral circulation to inflamed liver and differentiated into Ly6C^hi^ macrophages during fibrogenesis, we explored whether the decrease in Ly6C^hi^ macrophages in the fibrotic liver after BM-MSC transplantation is caused by preventing Ly6C^hi^ monocytes from recruiting into the liver. For this purpose, the proportion of CD11B^hi^F4/80^int^ monocytes/macrophages in the fibrotic liver was examined by FCM analysis. Minimal alteration in the proportion of CD11B^hi^F4/80^int^ cells was detected after BM-MSC transplantation (Fig. 3A, B). Consistently, the mRNA expression levels of *ccl2*, *ccr2*, and *cd11b* (myeloid lineage markers) were not changed in the fibrotic livers upon BM-MSC transplantation (Fig. 3C). For further clarification, an adoptive transfer assay was performed with cognate BM-MSCs and bone marrow-derived Ly6C^hi^ monocytes in a CCR2-deficient (CCR2^−^^/^^−^) mouse strain in an attempt to exclude the interference of endogenous bone marrow-derived Ly6C^hi^ monocytes. For this procedure, BM-MSCs and FACS-sorted bone marrow-derived CD135^−^CD117^−^CD115^+^Ly6C^hi^ monocytes (Fig. S7) were co-transferred into the fibrotic CCR2^−/^^−^ mice through the splenic pathway. Control fibrotic CCR2^−/−^ mice received CD135^−^CD117^−^CD115^+^Ly6C^hi^ monocytes alone. CD11B^hi^F4/80^int^ cells from the fibrotic liver were examined by FCM analysis (Fig. 3D). Expectedly, the proportion of CD11B^hi^F4/80^int^ monocytes/macrophages was not significantly decreased in the experimental group (Fig. 3E, F). These observations suggested that BM-MSCs did not have an inhibitory effect on the recruitment of Ly6C^hi^ monocytes into the injured liver, but the conversion of Ly6C^hi^ to Ly6C^lo^ macrophages in the fibrotic liver may be directly regulated by BM-MSCs.

### BM-MSCs regulate Ly6C^hi^/Ly6C^lo^ macrophage conversion by IL-4 and IL-10

**Fig. 4:**
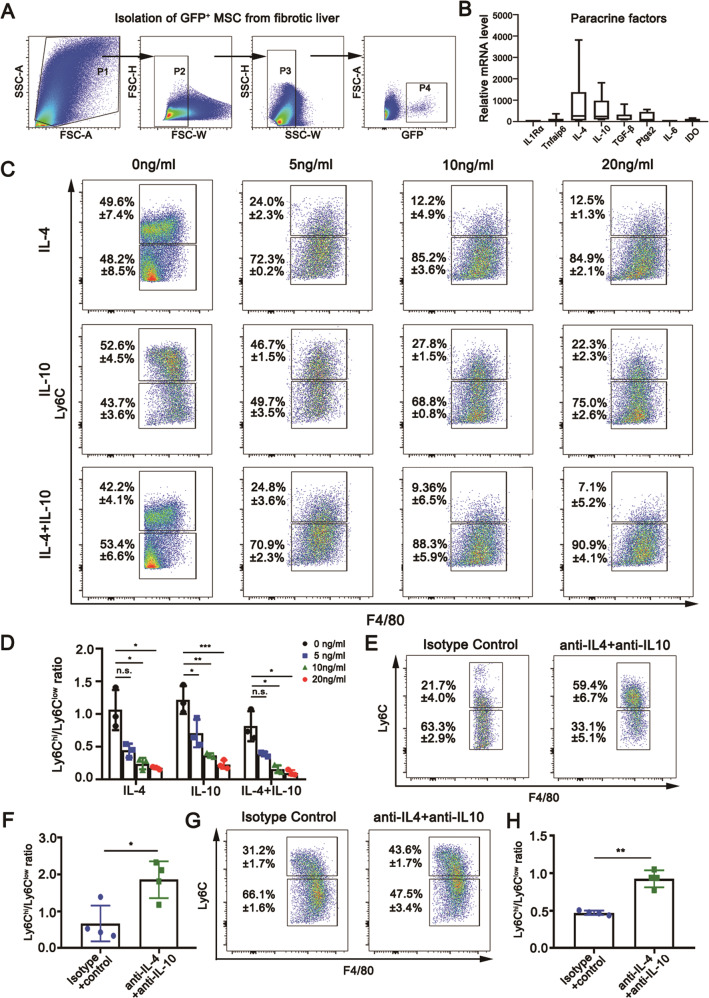
Examination of the phenotypic switch from Ly6C^hi^ to Ly6C^lo^ macrophages regulated by BM-MSCs. **A** Gating strategy for the isolation of GFP^+^BM-MSCs from fibrotic livers. **B** Upregulation of transcriptional expression levels of immunomodulatory factors in FACS-sorted GFP^+^BM-MSCs from fibrotic livers by quantitative real-time PCR (*n* = 6). **C** and **D** FCM analysis showing the stimulatory effects of IL4 and IL10 on the phenotypic switch of intrahepatic Ly6C^hi^/Ly6C^lo^ macrophages in a dose-dependent manner (*n* = 3). **E** and **F** FCM analysis showing the inhibitory effects of anti-IL-4 and anti-IL-10 antibodies on IL4/IL10-promoted phenotypic switch of intrahepatic Ly6C^hi^/Ly6C^lo^ macrophages. IgG2b isotype was used as a control. **G** and **H** The anti-IL-4 (0.6 μg/ml) and anti-IL-10 (1 μg/ml) antibodies and 10% ILCM (v/v) were added in BM-MSCs and Ly6C^hi^ macrophages coculture system. FCM analysis showed the direct effect of IL4 and IL10 secreted by BM-MSCs on the phenotypic switch of Ly6C^hi^/Ly6C^lo^ macrophages. Bars = means ± SD. Statistical evaluation of two groups was performed using an independent Student t-test. Statistical evaluation of multiple groups was performed using one-way ANOVA with posthoc LSD test; n.s. *p* > 0.05, *p < 0.05, **p < 0.01, ***p < 0.001.

BM-MSCs possess immunomodulatory activities by secreting immunoregulatory factors, we next explored whether the immunoregulatory factors potentially regulate Ly6C^hi^/Ly6C^lo^ macrophage conversion. The expression levels of IL-4, IL-10, TGF-β, PGE2, TSG-6, IL1Rα, IL-6, and IDO in transplanted BM-MSCs under the fibrotic liver microenvironment were initially examined by qPCR. For this procedure, GFP^+^BM-MSCs derived from C57BL/6-Tg (CAG-EGFP)1Osb/J strain were transplanted into the fibrotic liver. After infusion for 48 h, GFP^+^BM-MSCs were retrieved from the fibrotic liver by FACS for qPCR analysis. The results showed that *il-4* and *il-10* mRNAs in the retrieved BM-MSCs increased by 840 and 530 times compared with those in unstimulated control GFP^+^BM-MSCs without undergoing transplantation, however, minimal change was detected in IL1Rα and IL-6 transcripts (Fig. 4A, B). These observations suggested that IL-4 and IL-10 may play a major role in the BM-MSC-promoted conversion of Ly6C^hi^ to Ly6C^lo^ macrophages. For clarification, an in vitro polarization assay was conducted by incubating fibrotic liver-derived CD45^+^Ly6G^-^CD11B^hi^F4/80^int^Ly6C^hi^ macrophages with different concentrations of recombinant mouse IL-4 and IL-10 proteins. As expected, both IL-4 and IL-10 remarkably promoted the Ly6C^hi^/Ly6C^lo^ macrophage conversion in a dose-dependent manner, and a maximal conversion was observed in a combination of IL-4 (20 ng/mL) with IL-10 (20 ng/mL) (Fig. 4C, D). This finding suggested the existence of a synergistic effect between IL-4 and IL-10. Accordingly, a significant increase in the ratio of Ly6C^hi^/Ly6C^lo^ macrophages was shown in BM-MSC-transplanted fibrotic liver treated with anti-IL-4 and anti-IL-10 neutralizing antibodies compared with that treated with isotype control IgG2b (Fig. 4E, F). Furthermore, we conducted a direct experiment functionally linking Ly6C^hi/lo^ macrophage plasticity and IL-4/IL-10 secretion through BM-MSCs. For this, BM-MSCs and Ly6C^hi^ macrophages were cocultured in the presence of anti-IL-4 and anti-IL-10 antibodies (for neutralizing the activity of IL-4 and IL-10) and injured liver conditional medium (ILCM, v/v; for inducing the production of IL-4 and IL-10). The changes in macrophage plasticity were examined by FCM analysis. The results showed that the phenotypic switch from Ly6C^hi^ to Ly6C^lo^ macrophages was significantly inhibited in the neutralization group treated with anti-IL-4 and anti-IL-10 antibodies compared with the control group treated with the isotype IgG (Fig. 4G, H). These results suggested that BM-MSCs regulated Ly6C^hi^/Ly6C^lo^ macrophage conversion largely through secreting IL-4 and IL-10 upon stimulation in the fibrotic liver microenvironment. Additionally, we explored the potential regulatory role of BM-MSCs in Ly6C^hi^/Ly6C^lo^ macrophage conversion by a cell–cell contact-dependent mechanism. For this, CD11B^hi^F4/80^int^ macrophages sorted from fibrotic livers were cocultured with equivalent BM-MSCs in the presence of inhibitors for three promising contact-dependent signaling pathways: Wnt, Notch, and Hedgehog. FCM analysis showed that the ratio of Ly6C^hi^/Ly6C^lo^ macrophages did not significantly decline in inhibitor-administered cocultures compared with that of the control coculture that received mock DMSO (Fig. S8). These observations largely exclude the potential regulatory role of BM-MSCs in the conversion of Ly6C^hi^ to Ly6C^lo^ macrophages in cell–cell contact manner.

### BM-MSCs undergo apoptosis in fibrotic liver after transplantation

**Fig. 5:**
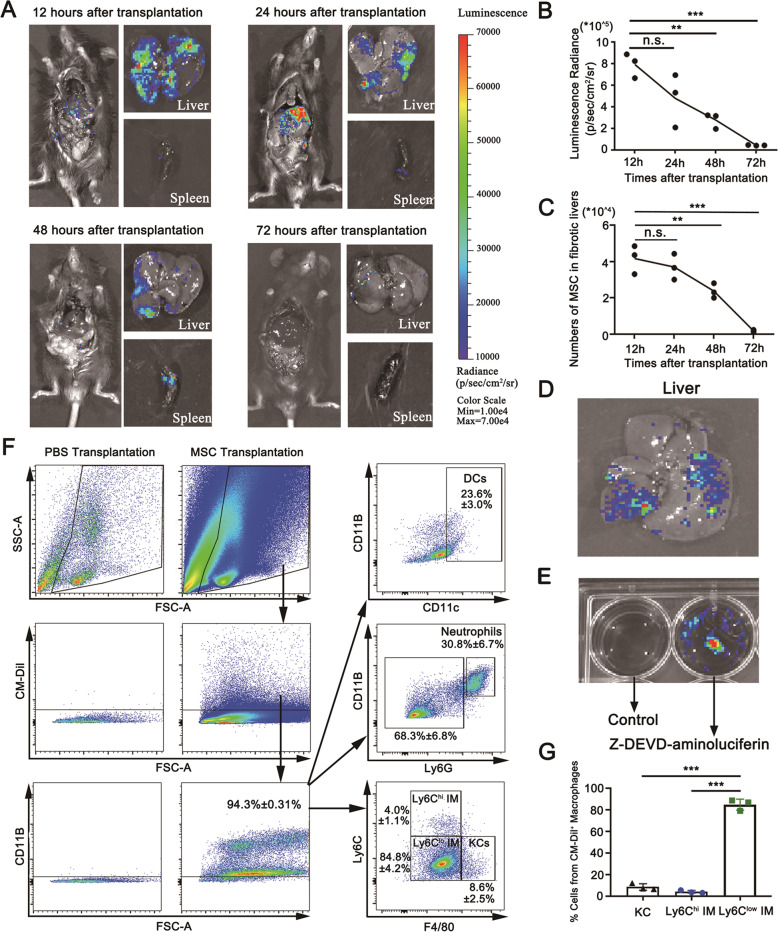
Cellular fate detection for transplanted BM-MSCs in fibrotic livers. **A** Distribution of Luc^+^ BM-MSCs transplanted into fibrotic mice was detected by in vivo imaging system (PerkinElmer). 1 × 10^6^ Luc^+^ BM-MSCs from the male donors were injected into female C57BL/6 J mice 24 h after the last CCl_4_ injection. Mice received D-luciferin (150 µg/kg) and were imaged at different times after infusion (*n* = 3). **B** Quantification of the luminescence radiance detected by an in vivo imaging system at different times after transplantation (*n* = 3). **C** Quantitative real-time PCR analysis for the transcriptional expression of Y chromosome-specific *ymt2/b* gene to illustrate the number of remaining BM-MSCs in fibrotic livers at different times after transplantation (*n* = 3). **D** 1 × 10^6^ Luc^+^ BM-MSCs were injected into C57BL/6 J mice 24 h after the last CCl_4_ injection. Mice received Z-DEVD-aminoluciferin (150 µg/kg) and were imaged at 4 h after infusion (*n* = 3). Z-DEVD-aminoluciferin is a modified firefly luciferase substrate that can be cleaved by caspase-3 to liberate aminoluciferin in apoptotic Luc^+^ BM-MSCs, latter of which is consumed by luciferase to generate a luminescent signal. **E** 1 × 10^6^ Luc^+^ BM-MSCs were treated by 50% ILCM (v/v) for 4 h, and Z-DEVD-aminoluciferin (50 µg/well) was used to detect apoptosis by an in vivo imaging system. **F** Representative flow cytometry plots of CM-Dil^+^ cells in the liver. The majority of CM-Dil^+^ signal was identified as Ly6C^lo^ macrophages. **G** Proportion of Kupffer cells (F4/80^hi^Ly6C^lo^), infiltrating Ly6C^hi^ macrophages (F4/80^int^Ly6C^hi^), and Ly6C^lo^ macrophages (F4/80^int^Ly6C^lo^) in the population of CM-Dil^+^ macrophages (*n* = 3). Bars = means ± SD; Statistical evaluation was performed using one-way ANOVA with posthoc LSD test; n.s. *p* > 0.05, *p < 0.05, **p < 0.01, ***p < 0.001.

To determine the fate of BM-MSCs in the fibrotic liver after transplantation, Luc^+^ BM-MSCs derived from C57BL/6J-TgN (Chicken-β-actin-LUC) ZLFILAS male mice were transplanted into syngeneic female fibrotic mice by spleen injection. Livers were harvested from recipient mice at different times after BM-MSC infusion, and the signals of Luc^+^ BM-MSCs were detected by an in vivo imaging system. The results showed that the Luc^+^ BM-MSC signals remarkably increased in the fibrotic liver at 12 h post-infusion followed by a drop in 72 h (Fig. 5A, B). The amount of Luc^+^ BM-MSCs in fibrotic livers was quantified via qPCR by detecting the copy number of the *Ymt2/b* gene specialized on the Y chromosome calculated by a standard curve. Consistent with the in vivo imaging result, the transplanted Luc^+^ BM-MSCs declined by 43% and 95% at 48 and 72 h, respectively, after infusion compared with the 12 h group (Fig. 5C). These observations suggested that BM-MSCs underwent rapid rejection through apoptotic cell death after transplantation. For clarification, an in vivo activation assay for apoptotic caspase-3 was examined in Luc^+^ BM-MSCs. In this case, the activation of caspase-3 was detected as luciferase activity by the administration of Z-DEVD-aminoluciferin. As expected, remarkable luminescence signals were detected in Luc^+^ BM-MSCs in the fibrotic liver, which indicated the occurrence of apoptosis accompanied with caspase-3 activation (Fig. 5D). Additionally, significant luminescence signals were detected after culturing Luc^+^ BM-MSCs with 50% injured liver conditional medium (ILCM, v/v) in vitro (Fig. 5E). To determine the fate of apoptotic BM-MSCs, the CM-Dil-stained BM-MSCs were transplanted into fibrotic livers and harvested 48 h after transplantation. Single-cell suspensions were prepared and stained by leukocyte markers for FCM analysis. The results showed that the CM-Dil^+^ signals were mostly found in CD11B^+^mononuclear cells, including macrophages, neutrophils, and dendritic cells. Further analysis showed that the majority of CM-Dil^+^ signals was found in CD11B^+^F4/80^+^ macrophages, whereas dendritic cells and neutrophils were the minority of CM-Dil^+^ signals. Thus, the infiltrating macrophages, rather than dendritic cells and neutrophils, played a major role in clearing the infused apoptotic BM-MSCs. There were 84.8% ± 4.2% CM-Dil^+^ signals in Ly6C^lo^ macrophages and 4.0% ± 1.1% CM-Dil^+^ signals in Ly6C^hi^ macrophages in the macrophage subset, which indicated that Ly6C^lo^ macrophages exerted a significant role in apoptotic cell clearance (Fig. 5F, G).

### Ly6C^lo^ macrophages upregulate MMP12 by engulfment of apoptotic bodies

**Fig. 6:**
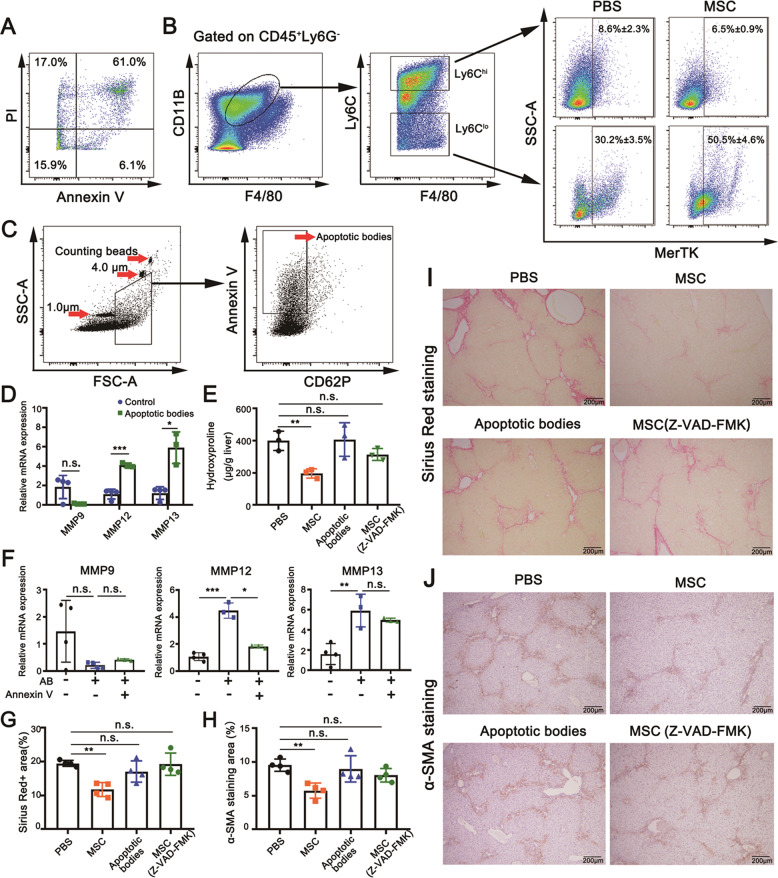
Examination of the engulfment of BM-MSC-derived apoptotic bodies by Ly6C^lo^ macrophages. **A** FCM analysis for apoptotic BM-MSCs induced by 50% ILCM (v/v) for 24 h through Annexin V and PI staining. **B** Representative FCM gating strategy used to identify differential MerTK expression in CD11b^hi^F4/80^int^Ly6C^hi^ and Ly6C^lo^ macrophages in the fibrotic liver after transplanting 1 × 10^6^ BM-MSCs or PBS control (*n* = 3). **C** Quantification of apoptotic bodies by FCM analysis. Calibration beads with diameters of 1.0 and 4.0 μm (internal reference beads) and counting beads with diameters of 7.0 μm were used to gate 1–5 μm-sized microvesicles. Apoptotic bodies were identified as AnnexinV^+^P62^−^. **D** Quantitative real-time PCR analysis for the transcriptional expression levels of matrix-degrading relevant *mmp9*, *mmp12*, and *mmp13* genes in BMDMs (1 × 10^6^) with engulfment of apoptotic bodies (3 × 10^7^) for 12 h (*n* = 3 and *n* = 4). **E** Quantification of the hydroxyproline content in livers after transplantation with PBS (control), BM-MSCs, BM-MSC-derived apoptotic bodies, and BM-MSCs pretreated with Z-VAD-FMK (40 μM) (*n* = 3). **F** Quantitative real-time PCR analysis for the transcriptional expression levels of *mmp9*, *mmp12*, and *mmp13* genes in BMDMs with engulfment of apoptotic bodies pretreated with Annexin V (1 μg/mL) for 12 h (*n* = 3 and *n* = 4). **G**, **I** Examination of the degree of liver fibrosis by staining tissue sections with Sirius Red (**I**) and semi-quantification through Image software (**G**) after mice were transplanted with PBS (control), BM-MSCs (1 × 10^6^), BM-MSC-derived apoptotic bodies (3 × 10^7^), and BM-MSCs (1 × 10^6^) pretreated with Z-VAD-FMK (40 μM) (*n* = 4). Scale bars, 200 μm. **H**, **J** Immunohistochemistry staining (**J**) and semi-quantification (**H**) of α-SMA in each group (**J**). The α-SMA^+^ areas were quantified from five random non-overlapping fields of each sample (*n* = 4). Scale bars, 200 μm. Bars = means ± SD. Statistical evaluation of two groups was performed using the independent Student *t-*test. Statistical evaluation of multiple groups was performed using one-way ANOVA with posthoc LSD test; n.s. *p* > 0.05, *p < 0.05, **p < 0.01, ***p < 0.001.

The above observations demonstrated the apoptotic fate of BM-MSCs in the fibrotic liver. For further support, the death of BM-MSCs was induced by in vitro stimulation with the ILCM. As expected, the proportions of apoptotic (Annexin V^+^ PI^+^ and Annexin V^+^PI^−^) and necrotic cells (Annexin V^−^ PI^+^) were detected to be 67.1% and 17.0% by FCM analysis (Fig. 6A). The majority of BM-MSCs underwent apoptosis upon inducers from the fibrotic liver. Thus, the apoptotic bodies derived from BM-MSCs in fibrotic livers may facilitate immunomodulation for fibrosis. Ly6C^lo^ macrophages are highly restorative for fibrosis by upregulating MMPs including MMP9, MMP12, and MMP13 to promote matrix degradation; this process is associated with the phagocytosis of Ly6C^lo^ macrophages that highly express PtdSer-dependent receptor tyrosine kinases (MerTK) for the transduction of signals from PtdSer. In support of this viewpoint, BM-MSCs were transplanted in the fibrotic liver, and the expression of MerTK was examined by FCM analysis. The results revealed that the expression of MerTK increased from 30%–50% in Ly6C^lo^ macrophages after BM-MSC infusion compared with the PBS control (Fig. 6B). Given that considerable BM-MSC-derived apoptotic bodies were engulfed by Ly6C^lo^ macrophages (Fig. 5F), we speculated that this engulfment initiated the upregulation of MMPs. For clarification, the primary bone marrow-derived Ly6C^hi^ monocytes were prepared and induced to CD11B^+^F4/80^+^Ly6C^lo^ mature phenotype (BMDMs) by L929 conditioned medium. The apoptosis of BM-MSCs was induced by ILCM, and the apoptotic bodies were isolated by FACS sorting (Fig. 6C). Subsequently, apoptotic bodies were added into the CD11B^+^F4/80^+^Ly6C^lo^ macrophages, and the transcriptional mRNA levels of *mmp9*, *mmp12*, and *mmp13* in cells were quantified by qPCR. The results showed that *mmp12* and *mmp13* were remarkably upregulated in response to apoptotic body stimulation (Fig. 6D). Furthermore, the apoptotic bodies pretreated with Annexin-V recombinant protein for the blockade of PtdSer impaired the phagocytosis. The results showed that the treatment significantly inhibited the expression of MMP12 (Fig. 6F). These outcomes suggested that MMP12 is likely a major effector in Ly6C^lo^ macrophage-mediated fibrosis resolution upon stimulation with BM-MSC-derived apoptotic bodies. For in vivo assay, BM-MSCs, apoptotic bodies, and BM-MSCs that were pretreated with Z-VAD-FMK were transplanted in fibrotic mice for liver fibrosis treatment. Expectedly, BM-MSC-infused livers showed low hydroxyproline levels and α-SMA staining area fractions compared with those of PBS-administered control groups. However, treatment with apoptotic bodies alone and BM-MSCs treated with Z-VAD-FMK showed no significant improvement in fibrosis compared with those of PBS-administered control groups (Fig. 6E, I, G, J, and H.).

### PtdSer−MerTK−ERK signaling axis is involved in MMP12 upregulation

**Fig. 7:**
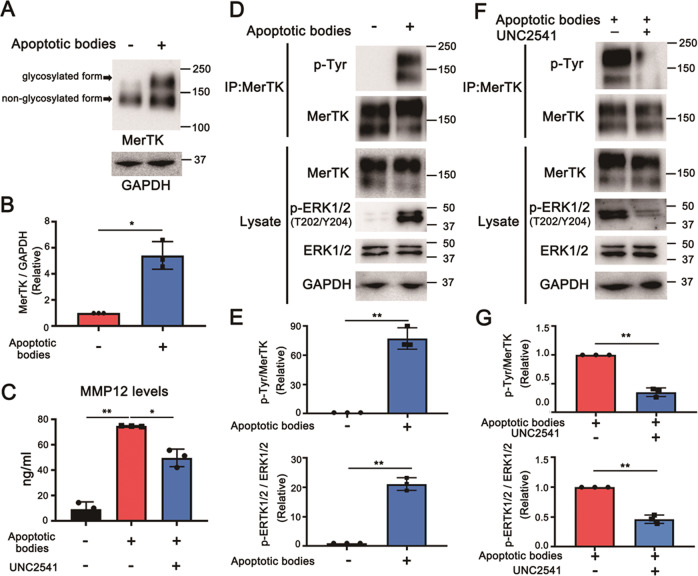
Examination of the activation of the MerTK signaling pathway in Ly6C^lo^ macrophages induced by BM-MSC-derived apoptotic bodies. **A**–**B** Detection and semi-quantification of MerTK proteins from BMDMs (1 × 10^6^) with or without stimulation of BM-MSC-derived apoptotic bodies (3 × 10^7^) for 30 min. Mouse MerTK is heavily glycosylated (15 N-glycosylation sites), the expression of MerTK with a 180 Kd glycosylated and 130 Kd non-glycosylated form were detected by Western blot (**A**) and ImageJ software (**B**) analyses (*n* = 3). **C** Examination of the inhibition of the MerTK signaling pathway in BMDMs by using UNC2541 inhibitor. MMP12 expression was detected as a downstream event for the activation of the MerTK signaling pathway. The secreted MMP12 protein was collected from the culture supernatant of BMDMs treated with mock PBS (control) or apoptotic bodies (3 × 10^7^) in combination with UNC2541 (1 μM) or without UNC2541 and detected by ELISA (*n* = 3). **D**–**E** Detection and semi-quantification of the phosphorylation of MerTK and ERK1/2 in BMDMs (1 × 10^6^) with or without stimulation of BM-MSC-derived apoptotic bodies (3 × 10^7^) by immunoprecipitation/Western blot (**D**) and ImageJ software (**E**) analyses (*n* = 3). The immunoprecipitation assay was performed by using anti-MerTK mAb, and phosphorylated MerTK was analyzed by Western blot assay with an HRP-conjugated anti-phosphotyrosine mAb. **F**, **G** Detection and semi-quantification of the phosphorylation of MerTK and ERK1/2 in BMDMs (1 × 10^6^) upon stimulation of BM-MSC-derived apoptotic bodies (3 × 10^7^) in combination with UNC2541 (1 μM) or without UNC2541 by immunoprecipitation/Western blot (**F**) and ImageJ software (**G**) analyses (*n* = 3). The immunoprecipitation and Western blot assays were performed followed by the methods as described in experiment D. Bars = ± SD. Statistical evaluation of two groups was performed using an independent Student *t*-test. Statistical evaluation of multiple groups was performed using one-way ANOVA with posthoc LSD test; *p < 0.05, **p < 0.01.

Finally, we explored whether the PtdSer-dependent MerTK signaling pathway is driven by BM-MSC-derived apoptotic bodies in the upregulation of MMP12 in Ly6C^lo^ macrophages. Western blot analysis showed that the MerTK was significantly upregulated in CD11B^+^F4/80^+^Ly6C^lo^ cells after incubation with apoptotic bodies (Fig. 7A, B). To clarify the activation of the MerTK signaling pathway in CD11B^+^F4/80^+^Ly6C^lo^ macrophages after being stimulated by the apoptotic bodies, the phosphorylation of MerTK was examined by immunoprecipitation and Western blot analysis. As expected, a phosphorylated 180 Kd band was clearly detected when CD11B^+^F4/80^+^Ly6C^lo^ macrophages were treated with apoptotic bodies. Furthermore, the downstream ERK1/2 was also significantly phosphorylated at Thr-202 and Tyr-204 (Fig. 7D, E). Meanwhile, the secretion of MMP12 was upregulated after stimulating CD11B^+^F4/80^+^Ly6C^lo^ macrophages by apoptotic bodies, as detected by ELISA (Fig. 7C). The correlation among MerTK, ERK1/2, and MMP12 was evaluated by utilizing UNC2541, an inhibitor for the phosphorylation of MerTK, in CD11B^+^F4/80^+^Ly6C^lo^ macrophages upon apoptotic-body stimulation. The results showed that inhibition of MerTK remarkably impaired the activation of ERK1/2 (Fig. 7F, G) and significantly decreased the expression of MMP12 (Fig. 7C). These results suggested the involvement of the PtdSer-MerTK-ERK1/2 signaling axis in apoptotic-body regulated MMP12 expression, which is crucial for matrix degradation in liver fibrosis.

## Discussion

Macrophages play pivotal roles in maintaining hepatic homeostasis and are closely associated with many liver diseases [21, 22]. Differential Ly6C expression is now used to identify the heterogeneous macrophage subsets responsible for fibrosis resolution in various tissues [22–25]. Regulation of phenotypic switch from Ly6C^hi^ to Ly6C^lo^ macrophages provides a promising therapeutic intervention for the treatment of liver fibrosis. However, it is not clear how this phenotypic conversion can be regulated. Here, we provided the first evidence that showed the dual regulatory roles of BM-MSCs in the attenuation of hepatic fibrosis by promoting Ly6C^hi^/Ly6C^lo^ macrophage switch through secreting antifibrogenic cytokines and actively initiating apoptotic cell death. Upon liver injury, the ratio of Ly6C^hi^/Ly6C^lo^ macrophages was markedly decreased after BM-MSCs transplantation. This decline was not caused by preventing Ly6C^hi^ monocytes recruitment, as confirmed by the adoptive transfer assays in CCR2^−/−^ mice. Thus, BM-MSCs directly led to a decreased ratio of Ly6C^hi^/Ly6C^lo^ macrophages in the fibrotic liver, which was evidenced by an in vitro coculture assay. qPCR analysis showed that *il-4* and *il-10* mRNAs were dramatically upregulated in BM-MSCs retrieved from the fibrotic liver, suggesting that these two cytokines played a major role in promoting Ly6C^hi^/Ly6C^lo^ macrophage conversion. This suggestion was validated by in vitro polarization assays via incubating Ly6C^hi^ macrophages with recombinant IL-4 and IL-10 cytokines and in vivo neutralization assays via administering anti-IL-4 and anti-IL-10 blocking antibodies into the BM-MSC-transplanted fibrotic liver. The expression levels of Nr4a1 and Cebpβ transcriptional factors were upregulated in intrahepatic CD11B^hi^F4/80^int^ cells during Ly6C^hi^/Ly6C^lo^ macrophage conversion. This result suggested that IL-4 and IL-10 may potentially regulate Ly6C^hi^/Ly6C^lo^ macrophage conversion by upregulating the Nr4a1 and Cebpβ transcriptional factors, which were previously recognized as two crucial regulators for Ly6C^hi^/Ly6C^lo^ macrophage polarization [26, 27]. Given that Ly6C^hi^ macrophages are a source of fibrogenic cytokines for promoting HSC activation and hepatic fibrogenesis. The conversion of Ly6C^hi^ to Ly6C^lo^ macrophages significantly downregulated the expression levels of Ly6C^hi^ macrophage-derived PDGF, TNF-α, and IL-1β in the fibrotic liver, which was accompanied with the reduced expression of HSC-derived α-SMA and Col1α1 and the reduction of liver fibrosis. These findings revealed that BM-MSCs could potently attenuate liver fibrosis by blocking the source of fibrogenic cytokines by promoting Ly6C^hi^/Ly6C^lo^ macrophage conversion at the early stage of transplantation.

Unexpectedly, we found that BM-MSCs experienced severe apoptosis and produced substantial apoptotic bodies in the fibrotic liver at 12−72 h post-transplantation. This observation was quite different from the inherent cognition that MSCs can be infused into injured tissues, where they survive, proliferate, and differentiate into multiple cell lineages [28]. Here, we proved that most apoptotic bodies from BM-MSCs were engulfed by Ly6C^lo^ macrophages in the fibrotic liver, as shown by in vivo tracking experiment using transgenic BM-MSCs from C57BL/6J-TgN mice with a chicken-β-actin-LUC reporter gene. The apoptotic bodies robustly promoted the expression of MMP12 in Ly6C^lo^ macrophages after phagocytosis. This reaction was dramatically attenuated by the treatment of cells with Annexin-V for the blockade of PtdSer, an “eat me” signal on apoptotic bodies that triggers the phagocytosis of phagocytes. Early studies have shown that the surface PtdSer on apoptotic bodies is detected by macrophage receptors with bridging protein [29], and prominent among the receptors is the Mer receptor tyrosine kinases, which constitute the Tryo3–Axl–Mer (TAM) family of receptors and is known for accelerating engulfment [30]. Thus, PtdSer is suggested to be closely associated with the MerTK-ERK signaling pathways. The potential involvement of the PtdSer-MerTK-ERK signaling axis in MMP12 upregulation was explored further. Expectedly, apoptotic bodies markedly stimulated the MerTK-ERK signaling pathway, as shown by the cascade phosphorylation of MerTK and its downstream ERK1/2 in Ly6C^lo^ macrophages. The activation of the MerTK-ERK pathway was accompanied with the secretion of MMP12. The correlation among MerTK, ERK1/2, and MMP12 was evidenced by utilizing a MerTK inhibitor in Ly6C^lo^ macrophages under stimulation with apoptotic bodies. These findings indicated that the PtdSer-MerTK-ERK1/2 signaling axis was involved in MMP12 expression, and it contributed to the key functional performance of restorative Ly6C^lo^ macrophages for ECM degradation in liver fibrosis. To our knowledge, this paper is the first report showing that BM-MSCs attenuated hepatic fibrosis by inducing restorative MMP12 from Ly6C^lo^ macrophages via activating the PtdSer-MerTK-ERK1/2 signaling pathway in an apoptosis-dependent manner.

**Fig. 8:**
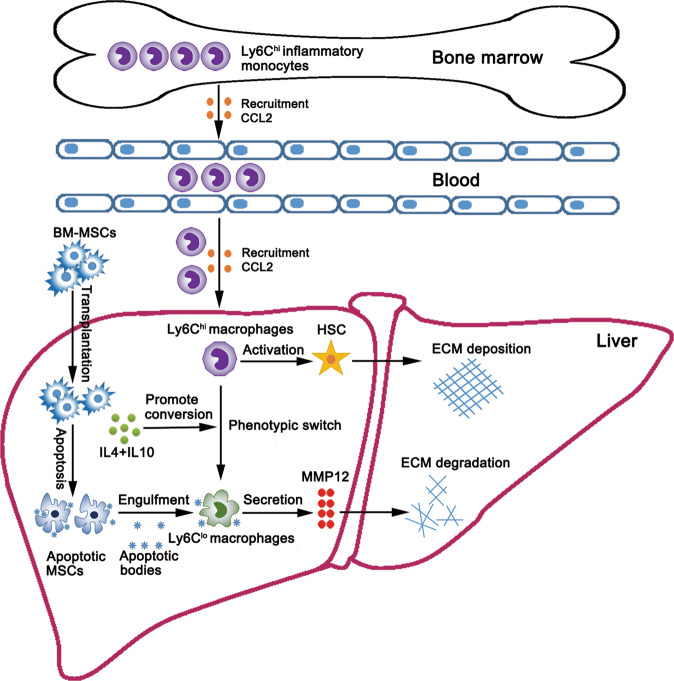
Schematic of how BM-MSCs may decrease liver fibrosis by targeting Ly6C^hi/lo^ macrophages through activating the cytokine-paracrine and apoptotic pathways. CCL2 secreted by the fibrotic liver promoted the recruitment of Ly6C^hi^ monocytes from bone marrow to the liver. BM-MSC transplantation promoted the conversion of Ly6C^hi^ to Ly6C^lo^ macrophages by secreting cytokines IL4 and IL10, which decreased the activation of HSCs. Meanwhile, BM-MSCs experienced severe apoptosis and produced substantial apoptotic bodies, which were engulfed by Ly6C^lo^ macrophages, leading to MMP12 release and accelerated extracellular matrix (ECM) degradation in CCL4-induced liver fibrosis.

In conclusion, we found that BM-MSCs attenuated hepatic fibrosis by converting Ly6C^hi^ to Ly6C^lo^ macrophages and promoting Ly6C^lo^ macrophage restoration through activating the antifibrogenic cytokine and apoptotic pathways (Fig. 8). Thus, this work uncovered a previously unrecognized dual regulatory function of BM-MSCs in liver fibrosis. The findings largely improved the current understanding of the cellular and molecular mechanisms underlying MSC-based therapy for liver fibrosis and injury repair.

## Materials and methods

### Animals and experimental models

C57BL/6 J and B6/JGpt-Ccr2^em8Cd6657^/Gpt were obtained from GemPharmatech. C57BL/6J-TgN (Chicken-β-actin-LUC) ZLFILAS mice were obtained from Shanghai Sciencelight Biology Science&Technology. C57BL/6-Tg (CAG-EGFP) 1Osb/J mice were obtained from the Jackson Laboratory. All mice were housed under standard conditions as previously described [20]. The 8- to 12-week-old and sex-matched mice were used for the experimental procedures. For liver fibrosis models, mice were intraperitoneally injected with CCl_4_ (1 mL/kg; 1:10 [v/v] in olive oil, Sangon Biotech) twice a week for 4 weeks. The animals were sacrificed 72 h after the final CCl_4_ injection, and serum and whole livers were collected for biochemical, histological, and molecular analyses as previously described [31]. For the transplantation experiment, mice were treated with CCl_4_ for 4 weeks with eight injections, and 1 × 10^6^ BM-MSCs were transplanted into mice 24 h after the final injection. The mice were then continually injected with CCl_4_ for another week until use. All animal experiments were performed in accordance with legal regulations and approved by a local ethics committee.

### Preparation and culture of BM-MSCs and BMDMs

Bone marrow-derived MSCs (BM-MSCs) were flushed from femurs or isolated from dissected fragments of femurs of C57BL/6 J mice, EGFP transgenic mice, and luciferase transgenic mice as described previously [31, 32]. The cells were cultured in Iscove’s modified Dulbecco’s medium (Gibco) supplemented with 10% FBS (Gibco) and 1% penicillin/streptomycin (Gibco) at 37 °C in 5% CO_2_. The medium was refreshed once a week, and BM-MSCs were passaged using 0.25% trypsin-EDTA (Gibco) at 80–90% confluence. BM-MSCs at passages 3−6 were used in experiments and identified by their lineage differentiation potential and surface phenotypic markers through flow cytometry analysis by using antibodies against CD29, CD44, CD105, Sca-1, CD31, CD34, CD45, CD11B, CD117, and CD135 [32, 33]. Bone marrow-derived macrophages (BMDMs) were generated from C57BL/6 J mice as described [34]. The whole bone marrow was cultured in DMEM (Gibco) supplemented with 10% FBS and 20% L929-conditioned medium for 2 days before removing non-adherent cells. The adherent cells were cultured for another 5 days, and this process yielded a macrophage population of >90% purity as detected by flow cytometry for CD11B and F4/80 [11].

### Isolation of hepatic nonparenchymal cells

Hepatic nonparenchymal cells (NPCs) were isolated using a modified protocol as described previously [35]. In brief, mice were anesthetized and fixed before surgical preparation. About 20 mL of PBS (10 mM, pH 7.0) was perfused through the inferior vena cava, followed by 5 mL of collagenase-pronase perfusion. Livers were harvested and digested in a digestive solution containing collagenase I (100 U/mL; Sigma), collagenase IV (100 U/mL; Sigma), Pronase E (25 μg/mL; Sigma), and DNase I (100 μg/mL; BioDuly) at 37 °C. After treatment for 40 min, a stopping solution containing DMEM, 10% FBS, and DNase I was added to inactivate the digestive enzymes. The livers were passed through a 40 μm cell strainer, and hepatocytes were removed by centrifugation at 50 g for 5 min at 4 °C. NPC suspension was collected at 500 g for 7 min at 4 °C, followed by separation with 30 and 70% Percoll (GE Healthcare) at 500 g for 25 min at room temperature. NPCs were collected from the interface between the 30 and 70% Percoll density cushion. Red blood cells were removed from NPCs by treatment with a lysis buffer (0.15 M NH_4_Cl, 10 mM KHCO_3_, and 0.1 mM EDTA in H_2_O) for 5 min on ice. The remaining NPC fraction was collected, counted, and prepared for flow cytometry and FACS sorting.

### Isolation of hepatic stellate cells

Hepatic stellate cells (HSCs) were prepared followed by a protocol as described previously [36]. Briefly, mice were anesthetized and underwent collagenase-pronase perfusion as described above. For quiescent HSC isolation, livers were harvested from healthy mice and digested in a digestive solution containing collagenase IV (100 U/mL; Sigma), Pronase E (mg/mL; Sigma), and DNase I (100 μg/mL; BioDuly) at 37 °C. For activated HSC isolation, livers were collected from fibrotic mice and digested in a digestive solution with collagenase I (100 U/mL; Sigma), collagenase IV (100 U/mL; Sigma), Pronase E (mg/mL; Sigma), and DNase I (100 μg/mL; BioDuly) at 37 °C. After treatment for 40 min, a stopping solution containing DMEM, 10% FBS, and DNase I was added to inactivate the digestive enzymes. Then, the livers were passed through a 40 μm cell strainer, and cell suspension from the digested livers was purified via 8.2% Nycodenz (Axis-shield, Oslo, Norway) gradient centrifugation at 580 g for 20 min at room temperature and filtered through a 40 μm cell strainer [31]. The obtained HSCs were cultured in plastic dishes with Iscove’s modified Dulbecco’s medium supplemented with 10% FBS and 1% penicillin/streptomycin at 37 °C in 5% CO_2_. The purity of HSCs was detected to be >99% following the method based on HSC-specific Vitamin A fluorescence as described [36].

### Histology analysis

For fibrosis examination, liver tissues were fixed with 10% neutral buffered formalin and embedded in paraffin. Subsequently, 5 μm tissue sections were dewaxed and rehydrated in decreasing concentrations of ethanol and stained with Sirius red (Sigma). The extent of fibrosis was captured under a microscope and quantified by Image software (Carl Zeiss, Germany). For immunohistochemistry analysis, 5 μm tissue sections were boiled in sodium citrate buffer (10 mM, pH 6.0) for 30 min for antigen retrieval and treated with 3% hydrogen peroxide to inhibit endogenous peroxidase activity. The sections were treated with 5% goat serum to block nonspecific binding sites and incubated with primary anti-α-SMA antibody (Abcam) at 4 °C overnight, followed by secondary HRP-conjugated goat anti-rabbit IgG antibody (1:500, Abcam) at 37 °C for 1 h. The color was developed using a DAB mixture (Beyotime). The extent of fibrosis was captured under a microscope (Carl Zeiss Axiostar plus, Germany) and semi-quantified by detecting the average optical density of the histochemical or immunohistochemical reactant area by ImageJ software as previously described [37].

### Flow cytometry and FACS analysis

For hepatic NPC analysis, cells were initially incubated with anti-rat Fc-receptor (CD16/32) for 10 min to exclude nonspecific staining. For phenotypic analysis of BM-MSCs and Ly6C^hi^/Ly6C^lo^ macrophages in cultures, the BM-MSCs of passages 3−10 and primary macrophages with specified treatment were harvested by 0.25% trypsin-EDTA digestion and stained with various antibodies (Table 1). Flow cytometry (FCM) analysis and fluorescent-activated cell sorting (FACS) were performed by using BD LSR Fortessa II systems and BD FACSAria II. FACS routinely yielded cell purity levels of over 95%. FCM analysis for apoptotic bodies was performed as previously described [38].

**Table1:** Key resources table.

Reagent or resource	Source	Identifier
***Antibodies***		
CD45, Clone 30-F11	eBioscience	Cat# 53-0451-82; RRID:AB_2848416
CD45, Clone 30-F11	eBioscience	Cat# 56-0451-82; RRID: AB_891454
CD11B, Clone M1/70	eBioscience	Cat# 45-0112-82; RRID: AB_953558
Ly6G, Clone RB6-8C5	eBioscience	Cat# 25-5931-82; RRID: AB_469663
Ly6C, Clone NK1.4	eBioscience	Cat# 12-5932-82; RRID:AB_10804510
Ly6C, Clone NK1.4	eBioscience	Cat# 62-5932-80; RRID: AB_2735067
F4/80, Clone BM8	eBioscience	Cat# 11-4801-82; RRID: AB_2637191
F4/80, Clone BM8	eBioscience	Cat# 17-4801-82; RRID: AB_2784648
CD11C, Clone N418	eBioscience	Cat# 63-0114-82; RRID:AB_2722930
CD11C, Clone HL3	BD Pharmingen™	Cat# 550261; RRID: AB_398460
CD3, Clone 17A2	eBioscience	Cat# 56-0032-82; RRID: AB_529507
NK1.1, Clone PK136	eBioscience	Cat# 12-5941-82; RRID: AB_466050
B220, Clone RA3-6B2	eBioscience	Cat# 62-0452-82; RRID: AB_2734992
CD115, Clone AFS98	eBioscience	Cat# 53-1152-82; RRID: AB_2016696
CD117, Clone 2B8	eBioscience	Cat# 62-1171-82; RRID:AB_2637141
CD117, Clone 2B8	eBioscience	Cat# 11-1171-81; RRID: AB_465185
CD135, Clone A2F10	eBioscience	Cat# 15-1351-82; RRID: AB_494219
CD135, Clone A2F10	eBioscience	Cat# 46-1351-80; RRID: AB_10733392
CD62P, Clone Psel.KO2.3	eBioscience	Cat# 12-0626-82; RRID:AB_1210863
CD29, Clone HMb1-1	eBioscience	Cat# 11-0291-80; RRID:AB_2572448
CD44, Clone IM7	eBioscience	Cat# 11-0441-81; RRID: AB_465044
CD105, Clone MJ7/18	eBioscience	Cat# 53-1051-80; RRID:AB_2815203
Sca-1, Clone D7	eBioscience	Cat# 45-5981-80; RRID: AB_914370
CD31, Clone 390	eBioscience	Cat# 46-0311-80; RRID:AB_1834430
CD34, Clone RAM34	eBioscience	Cat# 11-0341-81; RRID: AB_465020
MerTK, Clone DS5MMER	eBioscience	Cat# 63-5751-82; RRID:AB_2688139
anti-rat Fc receptor (CD16/32), Clone 2.4G2	BD Pharmingen™	Cat# 553140; RRID: AB_394655
anti-alpha smooth muscle actin Rabbit mAb	Abcam	Cat# ab5694; RRID: AB_2223021
anti-MerTK Rabbit mAb, Clone EPR17534-139	Abcam	Cat# ab184086
GAPDH Monoclonal Antibody, Clone ZG003	Invitrogen	Cat# 39-8600; RRID: AB_2533438
p44/42 MAPK (Erk1/2) Rabbit mAb	CST	Cat# 4695; RRID:AB_390779
Phospho-p44/42 MAPK (Erk1/2) Rabbit mAb	CST	Cat# 4370; RRID: AB_2315112
p-Tyr mouse mAb	PTM Bio	Cat# PTM-701
Goat anti Rabbit IgG H&L Polyclonal antibody, HRP conjugated	Abcam	Cat# ab6721; RRID:AB_955447
Goat anti-Mouse IgG H&L Secondary Antibody	Invitrogen	Cat# 31430; RRID: AB_228307
IL4, Clone 30340	Invitrogen	Cat# MA5-23722;RRID: AB_2609637
IL10, Clone JES5-16E3	Invitrogen	Cat# 16-7101-85; RRID: AB_469225
Rat IgG2b Isotype Control	Invitrogen	Cat# 02-9288; RRID: AB_2532966
***Chemicals, Peptide, and Recombinant Proteins***		
Annexin V (Ann-V) purified recombinant protein	Invitrogen	Cat# BMS306
Recombinant Mouse IL4	Peprotech	Cat# 214-14; RRID: AB_2609637
Recombinant Mouse IL10	R&D systems	Cat# 417-ML
Recombinant Mouse TGF-beta 1 Protein	R&D systems	Cat# 7666-MB
Recombinant Mouse Insulin R/CD220 Protein	R&D systems	Cat# 7544-MR
Vismodegib	Topscience	Cat# T2590
iCRT3	Topscience	Cat# T4302
Semagacestat	Topscience	Cat# T6125
UNC2541	Topscience	Cat# T17205
Z-VAD-FMK	MCE	Cat# HY-16658B
I collagenase	Sigma	Cat# C0130
II Collagenase	Sigma	Cat# C6885
IV collagenase	Sigma	Cat# C5138
Pronase E	Sigma	Cat# 1.07433
DNase I	BioDuly	Cat# E0046
DAPI	Sigma	Cat# D5942
IMDM	Gibco	Cat# 12200036
DMEM	Gibco	Cat# 12800017
FBS	Gibco	Cat# 10091155
Percoll	GE Healthcare	Cat# 17-0891-01
Nycodenz	Axis-shield	Cat# 1002424
BeyoGel™SDS-PAGE Precast Gel (4%-15%)	Beyotime Biotechnology	Cat# P0057A
Trizol Reagent	Invitrogen	Cat# 15596026
iScript^TM^ RT-qPCR Sample Preparation Reagent	Bio-Rad	Cat# 170-8898
D-Luciferin	Shanghai Sciencelight Biology Science&Technology	Cat#luc001
Z-DEVD-aminoluciferin	Promega	Cat# P1782
CellTracker™ CM-DiI Dye	Beyotime Biotechnology	Cat# C7000
Sirius red	Sigma	Cat#365548 CAS: 2610-10-8
Oil Red O	Sigma	Cat# O0625 CAS: 1320-06-5
Toluidine blue	Sigma	Cat# 89640 CAS: 6586-04-5
Alizarin Red S	Sigma	Cat# A5533 CAS: 130-22-3
olive oil	Sangon Biotech	Cat# A502795; CAS: 8001-25-0
***Critical Commercial Assays***		
MMP12 Elisa Kit	Sangon Biotech	Cat# D721034
BCA protein assay kit	Beyotime Biotechnology	Cat# P0465S
123count eBeads™ Counting Beads	Invitrogen	Cat# 01-1234-42
Flow Cytometry Size Calibration Kit	Invitrogen	Cat# F13838
Hydroxyproline assay kit	NanJing Jiancheng Bioengineering Institute	Cat# A030-2-1
Alanine aminotransferase Assay Kit	NanJing Jiancheng Bioengineering Institute	Cat# C009-2
Dead Cell Apoptosis Kit with Annexin V FITC and PI	Invitrogen	Cat# V13242
DAB Horseradish Peroxidase Color Development Kit	Beyotime	Cat# P0202
***Experimental Models: Cell lines***		
L929	ATCC	Cat# CCL-1^TM^
***Experimental Models: Organisms/Strains***		
C57BL/6 J	GemPharmatech	Cat# N000013
C57B6/JGpt-Ccr2^em8Cd6657^/Gpt	GemPharmatech	Cat# T006112
C57BL/6J-TgN (Chicken-β-actin-LUC) ZLFILAS	Sciencelight Biology Science&Technology	N/A
C57BL/6-Tg (CAG-EGFP)1Osb/J	The Jackson Laboratory	3291
***Software and Algorithms***		
GraphPad Prism 8	Graphpad Software	https://www.graphpad.com
Flowjo v10.5.0	Flowjo	https://www.flowjo.com
ImageJ	ImageJ software	https://imagej.nih.gov/ij
Image software	Carl Zeiss, Germany	N/A

### Quantitative real-time PCR

Total RNA of liver tissues or cultured cells was prepared using Trizol reagent (Invitrogen) or iScript^TM^ RT-qPCR Sample Preparation Reagent (Bio-Rad) according to the manufacturer’s protocols. About 500 ng of RNA was reverse-transcribed into cDNA using the SuperScript III kit (Invitrogen). The PCR experiments were performed in a total volume of 10 μL by using an iTaq Universal SYBR Green Supermix (Bio-Rad). Relative expression levels were calculated using 2^−Δ cycle threshold^ and 2^−ΔΔ cycle threshold^ methods with GAPDH for normalization. The transcript abundance of the target genes was analyzed through quantitative real-time PCR (qRT-PCR) on a CFX Connect Real-Time PCR Detection System (Bio-Rad) as previously described [20]. The primers for quantitative real-time PCR are listed in Supplemental Table.

### Assay for IL-4/IL-10-regulated Ly6C^hi^/Ly6C^low^ macrophage conversion

The regulatory role of IL-4 and IL-10 in the phenotypic switch of Ly6C^hi^/Ly6C^low^ macrophages was evaluated by in vivo neutralization and in vitro stimulation assays. For in vivo neutralization of IL-4 and IL-10, fibrotic mice were intraperitoneally administered with rat anti-mouse IL-4 (anti-IL-4) Ab (16 mg/kg, Invitrogen) and anti-IL-10 Ab (16 mg/kg, Invitrogen) twice at 12 and 24 h after injury induction. In parallel, non-related rat isotype Ab (IgG2b, 16 mg/kg) was used as control. For in vitro stimulation assay, CD11B^hi^F4/80^int^Ly6C^hi^ macrophages were sorted from the fibrotic liver by FACS and cultured without (control) or with different concentrations of recombinant mouse IL-4 and recombinant mouse IL-10 (5, 10, and 20 ng/mL) for 36 h, followed by FCM analysis.

### Imaging assay

After transplantation with Luc^+^ BM-MSCs (1 × 10^6^), mice were i.p. injected with D-luciferin (150 µg/kg body weight) for 15 min before sacrificing. Liver and spleen tissues were collected and imaged using an IVISSpectrum (PerkinElmer) imaging system for 1 min. Imaging data were analyzed and quantified with Living Image Software. The strength of the cellular signal was indicated by the spectrum. To detect apoptotic Luc^+^ BM-MSCs in the liver, mice were injected i.p. with VivoGlo Caspase-3/−7 substrate Z-DEVD Aminoluciferine (Promega; 300 µg/kg body weight) for 1–5 min and imaged using the IVISSpectrum imaging system. For in vitro assay, Luc^+^ BM-MSCs were pre-treated with 50% injured liver conditional medium (ILCM; v/v) for 4 h with or without Z-DEVD-aminoluciferin (50 µg/well) and imaged using the IVISSpectrum (PerkinElmer) imaging system for 1–5 min.

### Preparation of injured liver conditional medium

ILCM was prepared as described previously [28]. In brief, hepatocytes were isolated from fibrotic mice by two-step collagenase perfusion. After treatment, the perfused liver was resected and passed through a 40 μm cell strainer. The hepatocytes were collected by centrifugation at 50 g for 5 min and cultured in IMDM supplemented with 10% FBS at 5 × 10^4^ cells/cm^2^. After 48 h, the supernatant was collected and passed through a 0.22 μm filter. The filtrate was finally defined as ILCM and stored in aliquots at −40 °C for future use.

### Preparation of apoptotic bodies

For apoptosis induction, the cultural BM-MSCs were treated with ILCM for 24 h, and apoptotic bodies were isolated by a filter system as previously described [38]. In brief, cell debris was removed after 300 g centrifugation, and the supernatant was filtered with 5 and 1 µm filters. Apoptotic body-sized extracellular vesicles were isolated by 2000 g centrifugation at 4 °C for 20 min and stained with anti-CD62P-PE antibody and Annexin-V-APC on ice for 15 min for FCM analysis. Apoptotic bodies were characterized as positive for Annexin-V staining and negative for anti-CD62P-PE staining. The 1–5 μm apoptotic body-sized extracellular vesicles were gated by calibration beads (7 μm, Invitrogen) and counting beads (1 and 4 μm, 123 count eBeads™, Invitrogen). The number of counting beads was calculated following the manufacturer’s instructions. The number of apoptotic bodies was calculated by the number of counting beads multiplied by the ratio of apoptotic body events to counting bead events in FCM plots.

### Coculture experiments

Coculture experiments were performed to evaluate the regulatory activity of BM-MSCs to inflammatory macrophages. CD45^+^Ly6G^−^CD11B^hi^F4/80^int^ macrophages were sorted from the fibrotic liver by FACS and cocultured with BM-MSCs (5 × 10^5^) for 36 h. Subsequently, macrophages were digested by 0.25% trypsin-EDTA for 5 min, followed by centrifugation at 300 × g for 5 min. For signaling pathway inhibition, Vismodegib (40 μM), iCRT3 (40 μM), and Semagacestat (40 μM) were added in the cocultures for 36 h, and the control coculture received mock DMSO for FCM analysis. The phenotypic changes of the macrophages were examined by FCM analysis.

### Immunoprecipitation and Western blot analysis

For immunoprecipitation (IP), macrophages (1 × 10^6^) were lysed with cell lysis buffer (Beyotime Biotechnology). The lysates were centrifuged at 10,000 rpm at 4 °C for 10 min, and the supernatants were incubated with rabbit anti-MerTK mAb (Abcam) at 4 °C overnight. Protein A agarose beads (Thermo Scientific) were incubated with the mixture for 4 h. The beads were washed three times with lysis buffer, mixed with loading buffer, and incubated at 95 °C for 10 min. After centrifugation, the proteins were subjected to Western blot analysis. For this procedure, the samples were separated by BeyoGel™ SDS-PAGE Precast Gel (4–15%) and transferred to polyvinylidene difluoride membrane (EMD Millipore). After incubation with 2% BSA for 2 h at room temperature, the membranes were incubated with the primary antibody in TBST with 0.5% BSA overnight at 4 °C and then incubated with secondary antibody for 1 h at room temperature. The image was detected on a gel imaging system (Tanon 4500).

### Statistical analysis

All data were presented as the mean ± SD of each group. Statistical analysis was performed using GraphPad Prism 8 software and SPSS. Statistical evaluation of two groups was performed using an independent Student *t*-test. Statistical evaluation of multiple groups was performed using one-way ANOVA with a posthoc LSD test. A value of *p* < 0.05 was considered statistically significant. The sample size in the studies was estimated as previously reported [39]. Grubbs criterion was used to exclude outliers from the analysis. The samples were randomly assigned to experimental groups. The investigator was blinded to the group allocation during the experiment and when assessing the outcome as far as possible.

## Supplementary information

Supplemental Figure 1

Supplemental Figure 2

Supplemental Figure 3

Supplemental Figure 4

Supplemental Figure 5

Supplemental Figure 6

Supplemental Figure 7

Supplemental Figure 8

Supplemental Figure Legends

Supplemental Table
