# Supplementary figures and images for: Mesenchymal stem cells attenuate liver fibrosis by targeting Ly6Chi/lo macrophages through activating the cytokine-paracrine and apoptotic pathways
Source: Cell Death Discov. 2021 Sep 13;7:239. doi: 10.1038/s41420-021-00584-z (PMC8437974; doi:10.1038/s41420-021-00584-z)

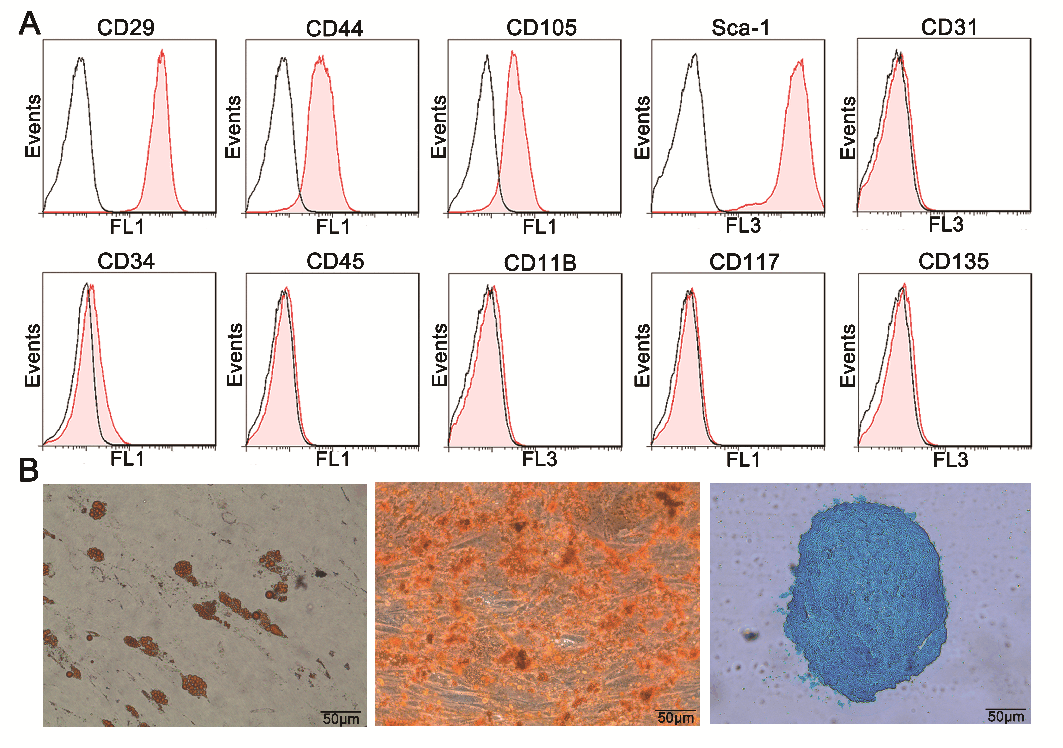

Supplement: Supplementary file 1 — Supplemental Figure 1 [file 41420_2021_584_MOESM1_ESM.png]

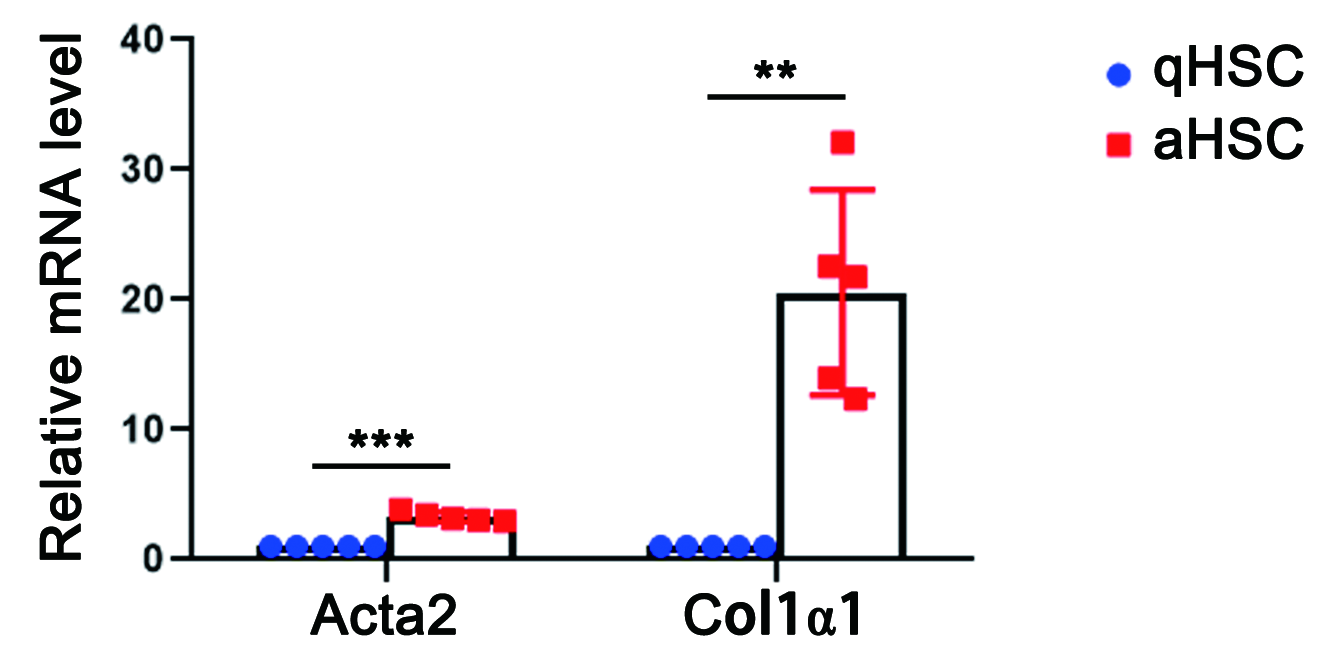

Supplement: Supplementary file 2 — Supplemental Figure 2 [file 41420_2021_584_MOESM2_ESM.tif]

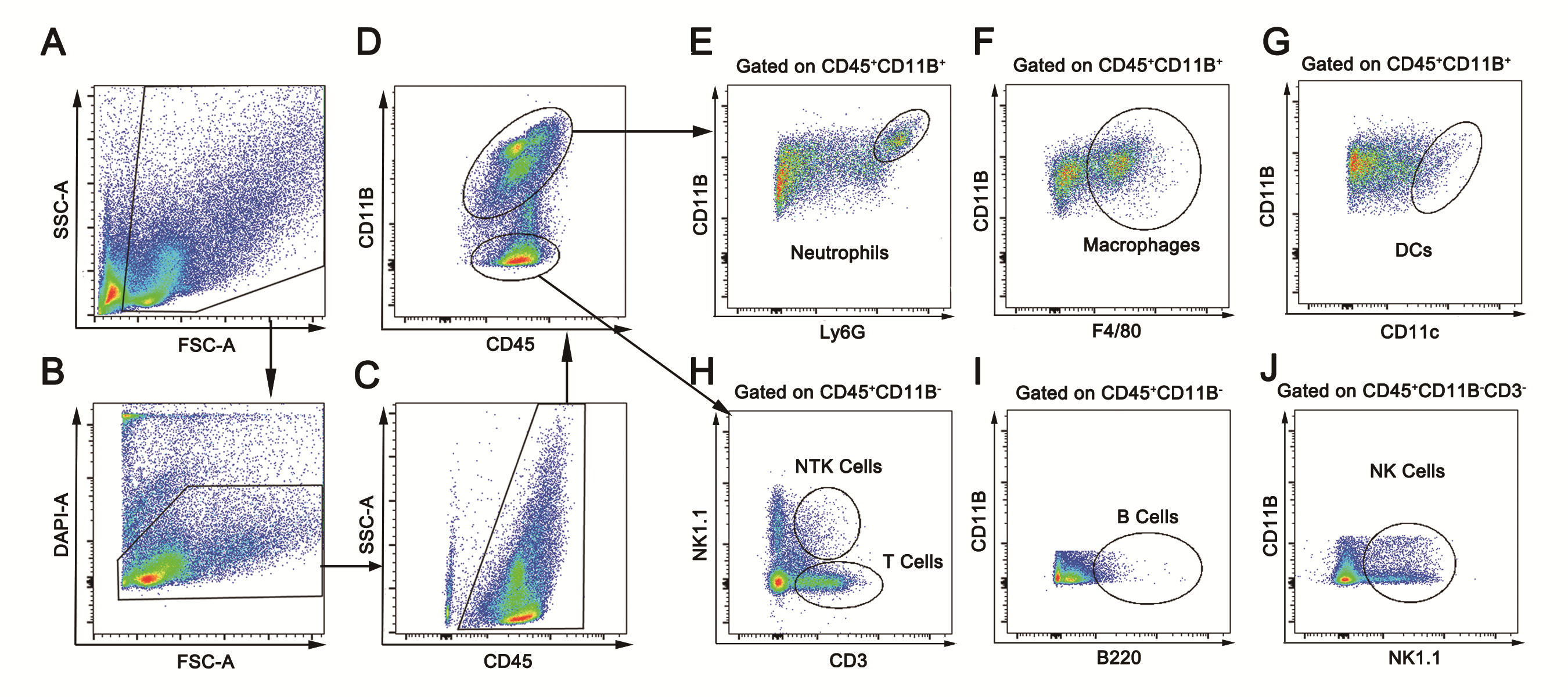

Supplement: Supplementary file 3 — Supplemental Figure 3 [file 41420_2021_584_MOESM3_ESM.png]

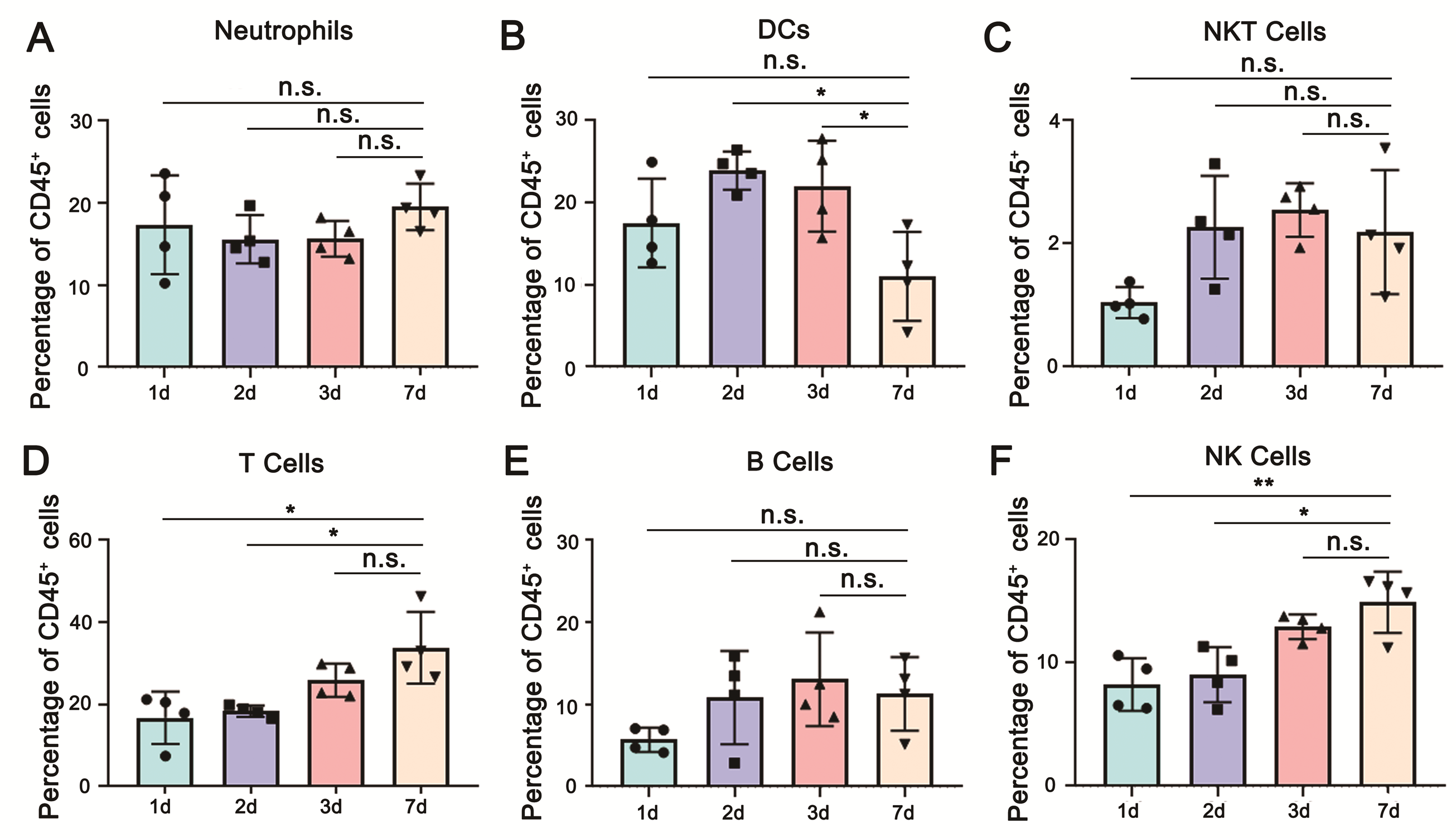

Supplement: Supplementary file 4 — Supplemental Figure 4 [file 41420_2021_584_MOESM4_ESM.png]

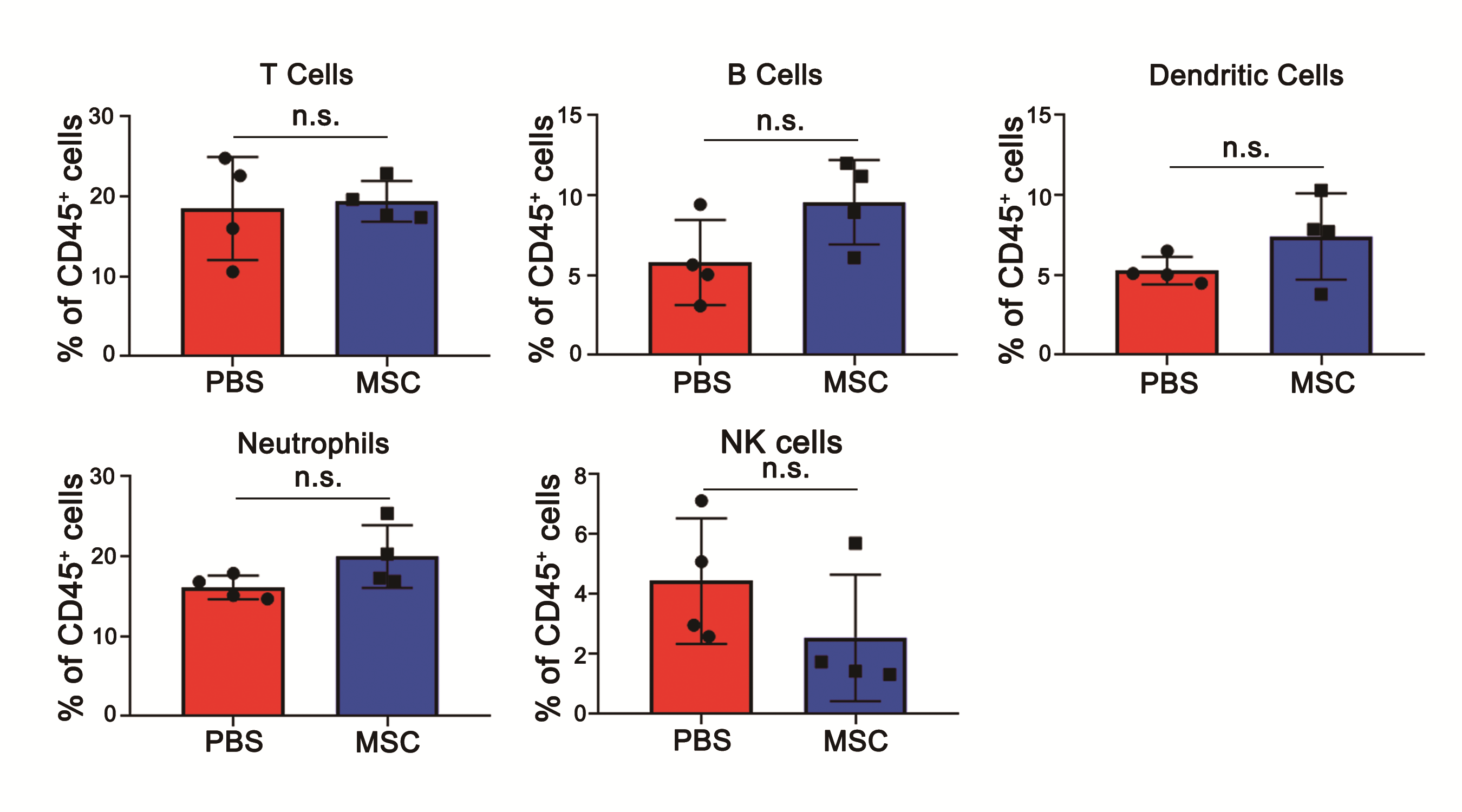

Supplement: Supplementary file 5 — Supplemental Figure 5 [file 41420_2021_584_MOESM5_ESM.png]

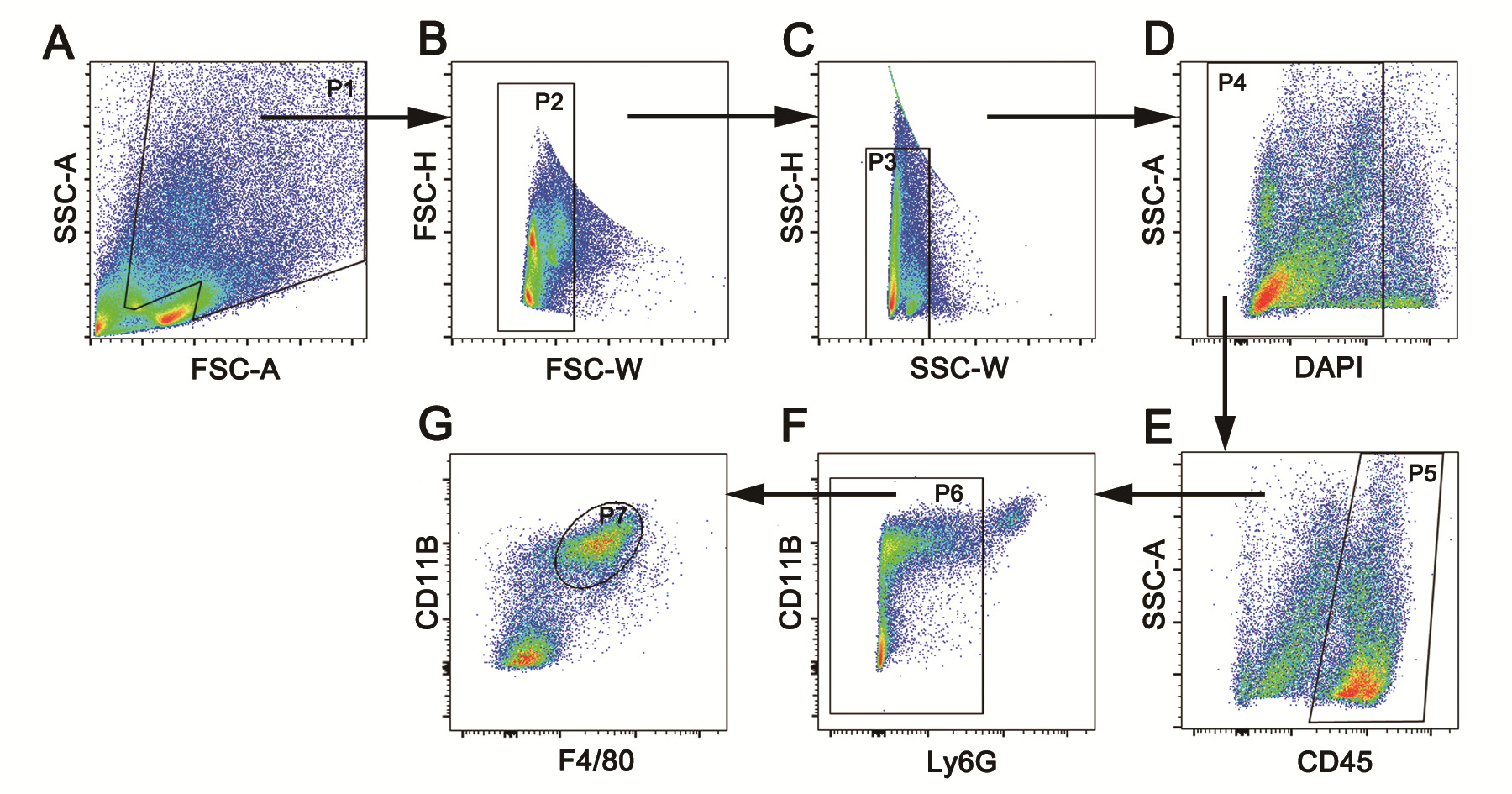

Supplement: Supplementary file 6 — Supplemental Figure 6 [file 41420_2021_584_MOESM6_ESM.png]

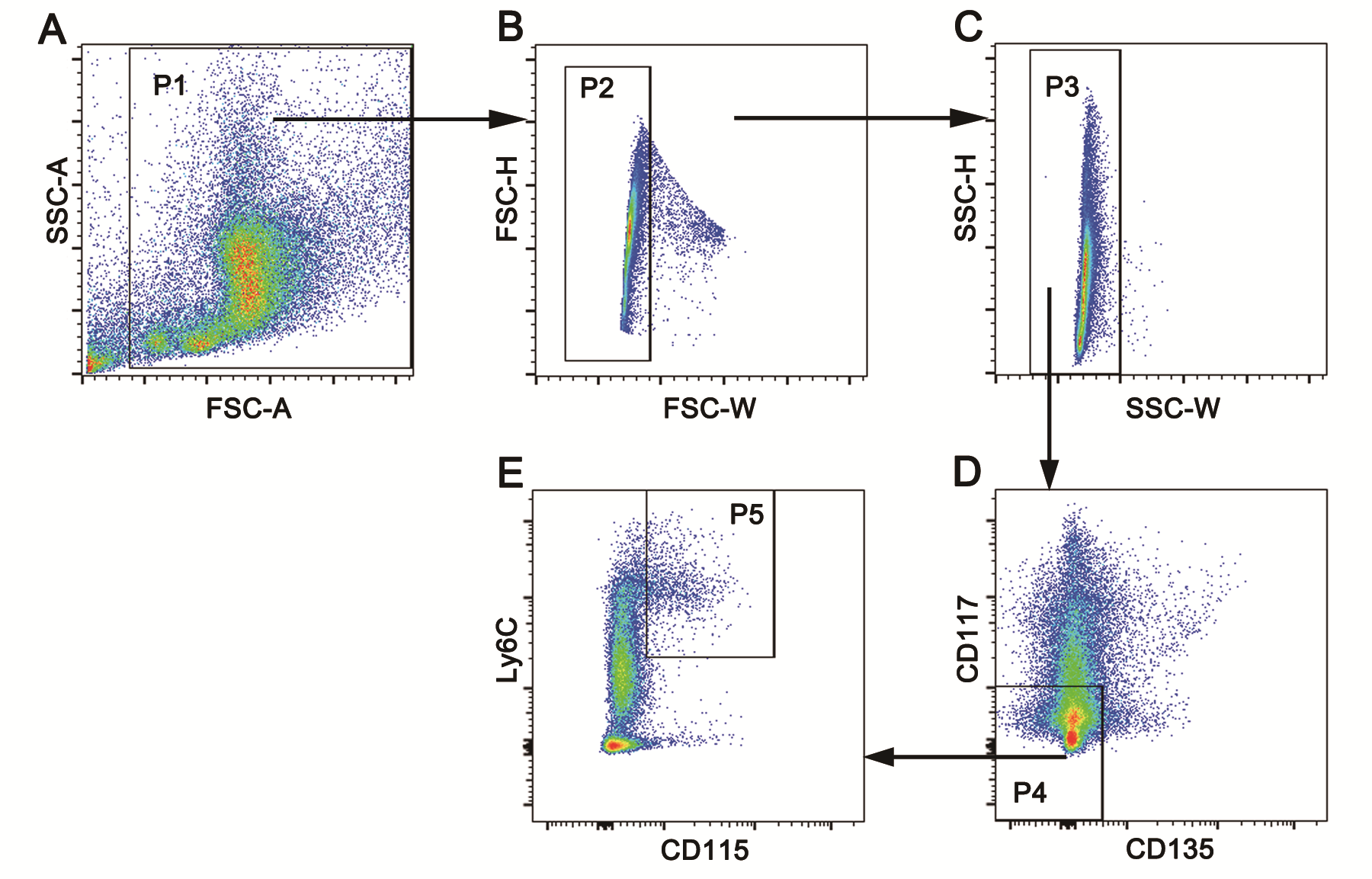

Supplement: Supplementary file 7 — Supplemental Figure 7 [file 41420_2021_584_MOESM7_ESM.png]

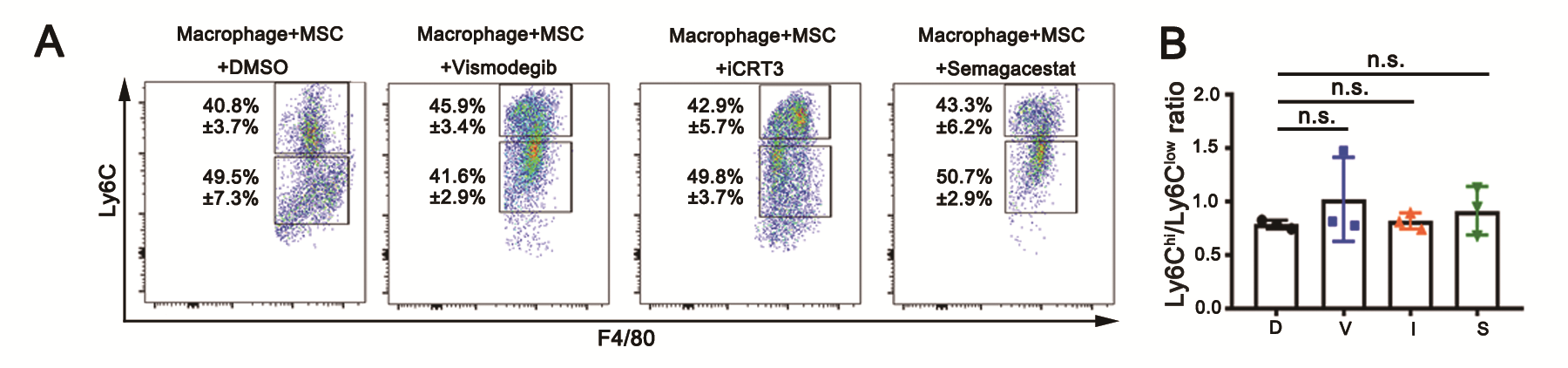

Supplement: Supplementary file 8 — Supplemental Figure 8 [file 41420_2021_584_MOESM8_ESM.png]
