# Supplementary material for: Mesenchymal stem cells attenuate liver fibrosis by targeting Ly6Chi/lo macrophages through activating the cytokine-paracrine and apoptotic pathways
Source: Cell Death Discov. 2021 Sep 13;7:239. doi: 10.1038/s41420-021-00584-z (PMC8437974; doi:10.1038/s41420-021-00584-z)
Supplement: Supplementary file 9 — Supplemental Figure Legends [file 41420_2021_584_MOESM9_ESM.docx]

**Supplemental Figure Legends**

**Figure S1. Characterization of mouse bone marrow-derived mesenchymal stem cells (BM-MSCs) by cell surface markers and differentiation capacities.** (A) FCM analysis of cell surface markers showed that the BM-MSCs were homogenously positive for mesenchymal lineage markers (CD29, CD44, and CD105) and progenitor cell markers (Sca-1) but negative for endothelial, myeloid and hematopoietic cell lineage-specific markers (CD31, CD34, CD45, CD11b, CD117, and CD135). (B) Examination of the differentiation capacities of BM-MSCs to adipocytes (left, stained with oil red), osteoblasts (middle, stained with alizarin red), and chondrocytes (right, stained with toluidine blue). Scale bars, 50 μm.

**Figure S2. Examination on the activation state of quiescent HSCs and activated HSC.** The quiescent HSCs were isolated from normal mouse liver and activated HSCs were isolated from fibrotic mouse liver. The method of isolating these two kinds of cells was described in detail in the Methods and Materials. For detection the activation of HSCs, relative expression levels of *acta2* and *col1α1* mRNA expression levels were examined by quantitative real-time PCR (n=5). Bars = means ± SD. Statistical evaluation of two groups was performed using independent Student t test. ^**^p<0.01, ^***^p<0.001.

**Figure S3. Gating strategies for the identification of various immunocyte subsets.** (A) Gating strategy was shown by FSC and SSC dot plot. (B) Viable cells were selected by 4'6-diamidino-2-phenylindole (DAPI) exclusion. (C) CD45-positive viable cells were gated. (D) Myeloid and lymphoid lineages were distinguished by the expression of CD11B. (E–J) Identification of neutrophils (E), macrophages (F), dendritic cells (G), T lymphocytes (H), natural killer T cells (H), B lymphocytes (I), and natural killer cells (J). Representative flow cytometry plots are shown.

**Figure S4. Examination of the proportion of various immunocytes in the liver at different time after CCl_4_ injection.** The proportion of various immunocytes including neutrophils (A), dendritic cells (B), NKT cells (C), T lymphocytes (D), B lymphocytes (E), and NK cells (F) were detected by FCM analysis at 1, 2, 3 (fibrogenesis stage), and 7 days (resolution stage) after the final CCl_4_ injection. Bars = means ± SD; Statistical evaluation was performed using one-way ANOVA with posthoc LSD test; n.s. p>0.05,

**Figure S5. Examination of the influence of BM-MSC infusion on the proportion of various immunocytes in the liver.** The proportion of various immunocytes including T lymphocytes, B lymphocytes, dendritic cells, neutrophils, NK cells were detected by FCM analysis at 7 days after BM-MSCs transplantation. Bars = means ± SD. Statistical evaluation of two groups was performed using independent Student t test; n.s. p>0.05

**Figure S6. Gating strategy for the identification of hepatic inflammatory macrophages.** (A) Gating strategy was shown by FSC and SSC dot plot. (B and C) Adhesion cells were excluded by FSC-H, FSC-W, SSC-H, and SSC-W. (D) Viable cells were selected by 4'6-diamidino-2-phenylindole (DAPI) exclusion. (E) CD45-positive viable cells were gated. (F) Neutrophils were identified as viable CD45^+^CD11b^+^ and Ly6G^+^ cells, and they were excluded from subsequent macrophage gating. (G) Hepatic inflammatory macrophages were identified as CD45^+^ Ly6G^-^CD11B^hi^ F4/80^int^ cells. Representative flow cytometry plots are shown.

**Figure S7. Gating strategy for the identification of bone marrow-derived CD115^+^CD11B^+^Ly6C^hi^ monocytes.** (A) Gating strategy was shown by FSC and SSC dot plot. (B and C) Adhesion cells were excluded by FSC-H, FSC-W, SSC-H, and SSC-W. (D-E) Ly6C^hi^ monocytes were identified as CD135^-^CD117^-^CD115^+^Ly6C^hi^ cells. Ly6C^hi^ monocytes were used for transfer experiment.

**Figure S8. Examination of the inhibitory effects of signaling pathway inhibitors on the phenotypic switch of hepatic Ly6C^hi^/Ly6C^lo^ macrophages.** (A-B) 5×10^5^  FACS-sorted CD11B^hi^F4/80^int^ macrophages from fibrotic livers were co-cultured with equivalent BM-MSCs for 36h in combination with Vismodegib, iCRT3, and Semagacestat to inhibit the Hedgehog, Wnt/β-catenin, and Notch signaling pathways, respectively. The ratio of Ly6C^hi^/Ly6C^lo^ macrophages was determined by FCM analysis (n=3). In figure (B), D, V, I, and S are abbreviations of experimental groups with CD11B^hi^F4/80^int^ macrophages + BM-MSCs + DMSO (control), CD11B^hi^F4/80^int^ macrophages + BM-MSCs + Vismodegib, CD11B^hi^F4/80^int^ macrophages + BM-MSCs + iCRT3, and CD11B^hi^F4/80^int^ macrophages + BM-MSCs + Semagacestat, respectively. (n=3). Bars = means ± SD; Statistical evaluation of multiple groups was performed using one-way ANOVA with posthoc LSD test; n.s. p>0.05
